# A direct interaction of cholesterol with the dopamine transporter prevents its out-to-inward transition

**DOI:** 10.1371/journal.pcbi.1005907

**Published:** 2018-01-12

**Authors:** Talia Zeppelin, Lucy Kate Ladefoged, Steffen Sinning, Xavier Periole, Birgit Schiøtt

**Affiliations:** 1 Department of Chemistry, Aarhus University, Aarhus C, Denmark; 2 Interdisciplinary Nanoscience Center, Aarhus University, Aarhus C, Denmark; 3 Translational Neuropsychiatry Unit, Department of Clinical Medicine, Aarhus University, Aarhus C, Denmark; University of Maryland School of Pharmacy, UNITED STATES

## Abstract

Monoamine transporters (MATs) carry out neurotransmitter reuptake from the synaptic cleft, a key step in neurotransmission, which is targeted in the treatment of neurological disorders. Cholesterol (CHOL), a major component of the synaptic plasma membrane, has been shown to exhibit a modulatory effect on MATs. Recent crystal structures of the dopamine transporter (DAT) revealed the presence of two conserved CHOL-like molecules, suggesting a functional protein-CHOL direct interaction. Here, we present extensive atomistic molecular dynamics (MD) simulations of DAT in an outward-facing conformation. In the absence of bound CHOL, DAT undergoes structural changes reflecting early events of dopamine transport: transition to an inward-facing conformation. In contrast, in the presence of bound CHOL, these conformational changes are inhibited, seemingly by an immobilization of the intracellular interface of transmembrane helix 1a and 5 by CHOL. We also provide evidence, from coarse grain MD simulations that the CHOL sites observed in the DAT crystal structures are preserved in all human monoamine transporters (dopamine, serotonin and norepinephrine), suggesting that our findings might extend to the entire family.

## Author summary

It has been revealed that the cellular membrane is an active contributor to biological processes occurring in and across it and can regulate the function of proteins embedded within it. Cholesterol (CHOL) plays a key role in these effects. Notably, CHOL affects neurotransmission. Neurotransmission occurs at the synapse and is central to a proper functioning of the human nervous system. In particular, monoamine transporters (MATs), key players in the process of stopping signaling, are primary targets for treatment of mental disorders. Herein, we address the intriguing mechanism by which the presence of CHOL affects the function of MATs. CHOL is known to modulate the re-uptake of neurotransmitters from the synapse but the mechanism is not known. We looked at the possibility of CHOL effecting MATs by a direct interaction rather than through its effect on the biophysical properties of the embedding membrane. We present clear data indicative of conserved CHOL binding sites on all human MATs and further show how CHOL prevents conformational changes at the intracellular end of transmembrane helix 5 in the human dopamine transporter (hDAT), which we are able to confirm is associated with the outward-to-inward transition of MATs.

## Introduction

Monoamine transporters (MATs) [1] encompass the dopamine (DAT), serotonin (SERT), and norepinephrine (NET) transporters and belong to the solute carrier 6 (SLC6) gene family [2]. MATs transport their respective neurotransmitters from the synaptic cleft back into the presynaptic neurons thereby ending neuronal signaling; a key function for neurotransmission that is directly linked to human wellbeing. Dysfunction of MATs has been associated with several psychiatric diseases such as depression [3], ADHD [4], Parkinson’s [4], and addiction [5], making MATs the primary targets for treatment of mental disorders [6].

**Fig 1:**
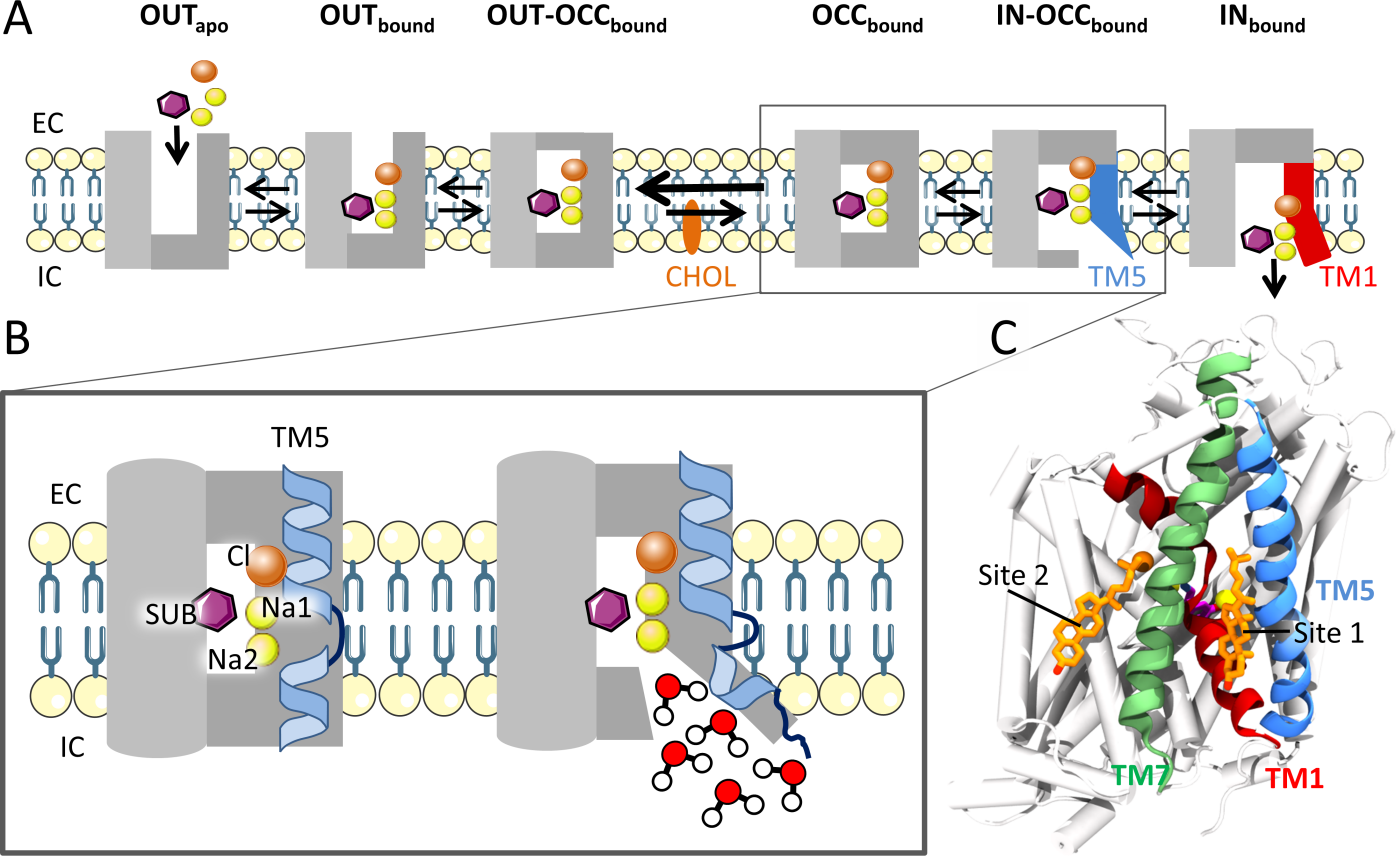
Schematic representation of the first half of the monoamine transport cycle. (A) The cycle shows the conformational states of the transporter while going from an outward-open to an inward-open conformation. The purple hexagon and the orange and yellow spheres represent the substrate and coordinating ions, respectively, and the orange ellipse represents CHOL. CHOL is thought to stabilize the outward-facing conformation of the transporter, which is emphasized by the arrows between the OUT-OCC_bound_ and OCC_bound_ states. The light grey represents the scaffold, while the dark grey indicates the moving segments of the protein, the bundle. TM5 is highlighted in blue and TM1a in red. (B) Close-up of the states OCC_bound_ and IN-OCC_bound_ with emphasis on the structural changes of TM5 between the two states. (C) hDAT with TM1, TM5 and TM7 highlighted together with the co-crystalized CHOL molecules at site 1 and 2 (orange sticks) as found in dDAT. Substrate and ions are colored as in panel A.

All MATs share a conserved structural motif consisting of 12 transmembrane (TM) helices that span the synaptic plasma membrane [2]. They function by the alternating-access mechanism in which the transporter follows a cycle interchanging between outward-facing (open to the extracellular, EC, side) and inward-facing (open to the intracellular, IC, side) conformations. The central substrate binding site is thus only accessible to one side of the membrane at a time [7] (Fig 1A). The sodium and chloride coordination sites (Na1, Na2, and Cl) are located adjacent to the substrate binding site. These ions are co-transported with the substrate during each transport cycle. Our focus herein is on the out-to-inward transition of the transport cycle (Fig 1B). Based on both experimental and modeling studies on MATs [8–14] and two bacterial homologues, LeuT [14–19] and MhsT [20], a transition mechanism has been suggested. From early structures of LeuT in the inward- and outward facing states, the rocking bundle model was originally proposed, in which the four helices, TM1, TM2, TM6, and TM7 act collectively as a bundle relative to the remaining helices which correspond to the scaffold [21]. Since then, the model has been refined, and a more substantial role of TM5 is now emerging [20,22].

The sequence of events leading to the out-to-inward transition are considered to be the closure of the thin gates on the EC side of the transporter (OUT-OCC_bound_), consisting of a salt bridge formed between R85-D476 and a hydrophobic lid established between Y156-F320 (hDAT numbering is used throughout the manuscript unless stated otherwise) [16,23], followed by a helix rearrangement resulting in a fully occluded state (OCC_bound_ in Fig 1A). Recent crystal structures of MhsT in the inward-facing occluded state (IN-OCC_bound_) showed IC unwinding of TM5, and the presence of an IC water cavity solvating the Na2 site (consisting of the coordinating residues G75, S422, D421, L418, and V78) which could possibly lead to Na^+^ release from this site [20] (Fig 1B). The final step of the transition to the inward-facing conformation is considered to occur through the breakage of the IC interaction network encompassing R445-E428, Y335-E428, and R60-D436 [24] and TM1a outward kinking that ultimately results in the release of the substrate and remaining ions [15]. Note that TM5 unwinding was not observed in the LeuT inward-facing apo structure, which suggests reformation of TM5 occurs prior to TM1a kinking [22].

Lipid rafts are ubiquitous micro-domains rich in cholesterol (CHOL) and sphingolipids implicated in membrane compartmentalization, organization and function [25,26]. They exist in many cell types and in neuronal cells in particular [27], where MATs have been demonstrated to associate with such domains [28–30]. Furthermore, CHOL depletion experiments have shown that lipid rafts both regulate DAT and SERT cell-surface expression [31–33] and modulate their activity [29,30]. CHOL has previously been shown to stabilize the outward-facing conformation of the MATs [34–36] and some studies suggest a direct CHOL/DAT interaction [37,38]. Additional data is however required to determine how and where this/these direct interactions occur.

Crystal structures of human SERT (hSERT) and *Drosophila melanogaster* DAT (dDAT) in the outward-facing conformation (OUT_bound_) have recently been solved [39–42] and CHOL-like molecules are found bound to the protein surface in all structures although at different locations. In dDAT, a CHOL molecule is consistently found at the interface of TM1a, TM5, and TM7 (site 1) and a CHOL analogue, cholesteryl hemisuccinate (CHS), is found at the interface between TM2 and TM7 in a subset of structures (site 2) (Fig 1C) [40,42]. Together with previous studies showing CHOL having a stabilizing effect on the outward-facing conformation on DAT and SERT [34–36] and that DAT may be regulated by a direct CHOL interaction [37,38], these bound CHOL molecules suggest a potential effect of CHOL at site 1 and 2 on DAT and possibly even MAT function. Gouaux and co-workers have postulated that CHOL at site 1 inhibits an out-to-inward transition by blocking TM1a outward kinking, since this movement is seen to be the largest when transitioning to the inward-facing conformation based on the LeuT crystal structures [15,39]. In the hSERT structure [41], a CHS molecule was found on the extracellular part of TM12. The relevance of this molecule to MAT function is not clear.

Here we use molecular dynamics (MD) simulations at multiple resolutions to elucidate the role of CHOL binding to MATs. We performed coarse grain (CG) MD simulations of hDAT, dDAT, hNET, and hSERT aiming at uncovering whether the two CHOL sites observed in the dDAT crystal structures and the single CHOL site found in the hSERT structures exist across MATs in a bilayer environment. Furthermore, we performed all-atom (AA) MD simulations of MATs with an emphasis on hDAT with and without bound CHOL molecules in order to shed light on the possible direct effect these have on the transport cycle. We show that one of the conserved CHOL binding sites on MATs has a strong effect on the out-to-inward transition of hDAT. A bound CHOL at that site acts by preventing early events that characterize the out-to-inward transition and thereby stabilizes hDAT in an outward facing conformation.

## Results

### Cholesterol binding sites

To explore the relevance of CHOL binding sites observed in the dDAT and hSERT crystal structures under near-physiological conditions for all MATs, we employed a 50 μs coarse grain (CG MD) simulation of each transporter (hSERT, hDAT, hNET and dDAT) embedded in a POPC:CHOL membrane bilayer at a 4:1 molecular ratio (see Table 1 for a simulation overview). CHOL exhaustively explores the TM section of the transporters by experiencing several hundreds of binding/unbinding events. We used a crystal structure for both hSERT and dDAT as well as a homology model for hDAT and hNET (see Methods for details).

**Table 1:** Overview of the AA and CGMD simulations performed.

	Name	Protein template or crystal structure (PDB ID)	Membrane composition	Number of repeats x simulation time (μs)
CG	*CG-dDAT*	dDAT (4XP1)	POPC:CHOL	1 x 50
	*CG-hDAT*	dDAT (4XP1)	POPC:CHOL	1 x 50
	*CG-hSERT*	hSERT (5I6X)	POPC:CHOL	1 x 50
	*CG-hNET*	dDAT (4XP1)	POPC:CHOL	1 x 50
AA	*AA-hDAT-wo-CHOL*	dDAT (4XP1)	POPC	5 x 1
	*AA-hDAT-w-CHOL*	dDAT (4XP1)	POPC + 2 CHOL	5 x 1
	*AA-hSERT*	hSERT (5I6X)	POPC	3 x 2

In the POPC:CHOL membrane a 4:1 molecular ratio was used.

**Fig 2:**
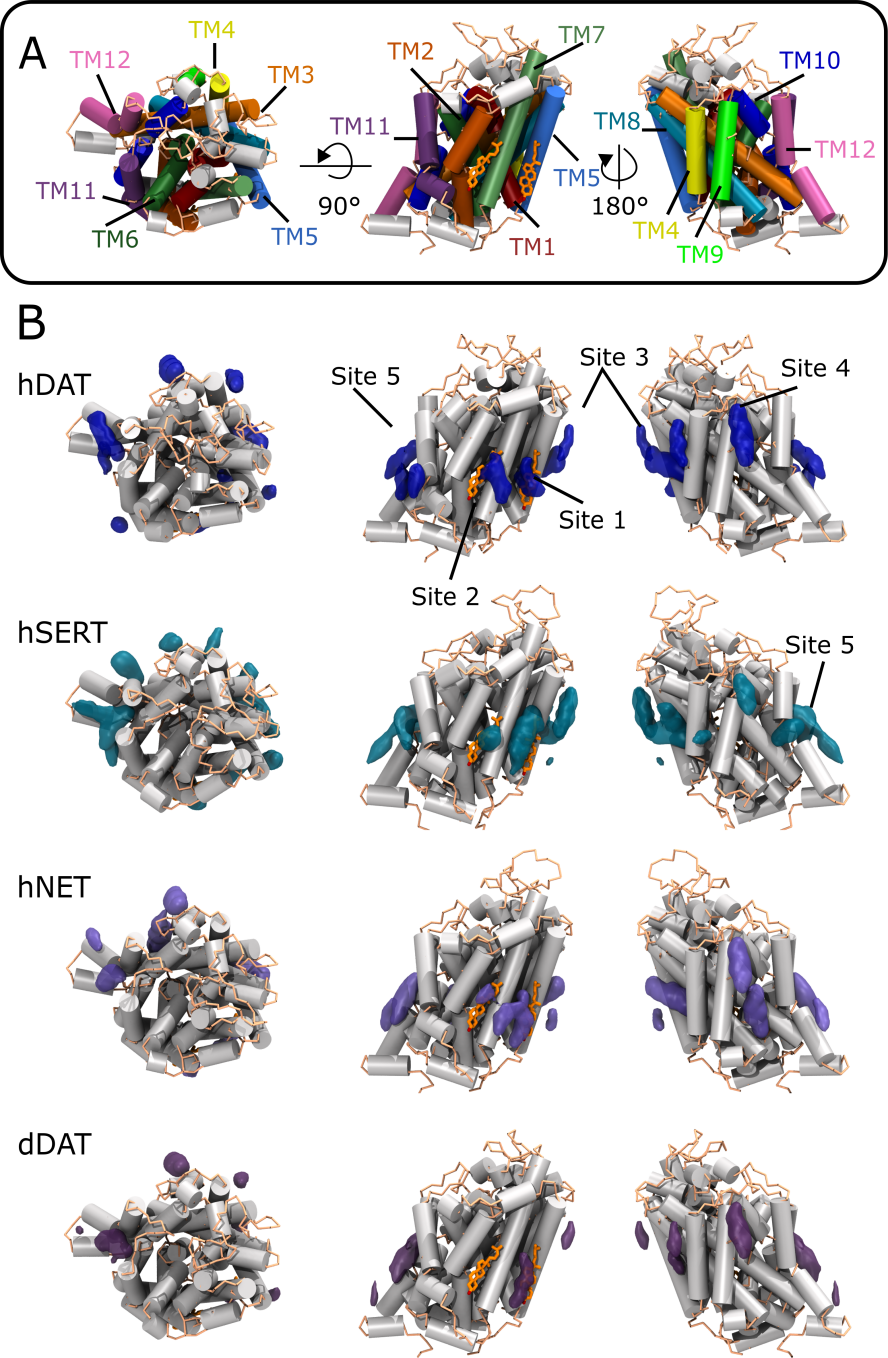
Cholesterol density maps at the surface of the monoamine transporters. (A) hDAT overview with the 12 TM helices highlighted. The two co-crystallized CHOL molecules observed in the dDAT crystal structures are superimposed on CG hDAT for emphasizing the location of site 1 and 2 (orange sticks). (B) CHOL occupancy maps for the four CG systems: hDAT, hSERT, hNET, and dDAT in the presence of 20% cholesterol. The maps depict an occupancy level at least 3 times higher than the values corresponding to the bulk region. The 5 conserved sites are indicated. Going from left to right, the structures are shown in topview (EC) and from two different sides (separated by a 180° rotation around the membrane normal) from within the membrane plane.

3D occupancy maps of CHOL were constructed from the CG MD simulations using the Volmap plugin in VMD [43]. The maps clearly indicate 5 preferential zones of CHOL interaction sites that are shared by all human MATs and dDAT (Fig 2, Table 2). This suggests a conserved pattern of CHOL binding sites across human MATs and possibly other species as well. All CHOL binding sites are located on the IC side of the lipid bilayer with the exception of site 4, which is located on the EC side (Table 2). Site 1 and 2 both share TM7 in their binding site and site 1 and 3 share TM5. Notably, the sites corresponding to the two co-crystallized CHOL/CHS molecules in dDAT, sites 1 and 2, are observed in the CG MD simulations for all MATs, albeit with varying degrees of occupancy (Fig 2 and Table 3).

**Table 2:** Location of the cholesterol binding sites conserved among MATs.

Cholesterol site	Location	Membrane side
Site 1	TM1a, TM5, TM7	IC
Site 2	TM7, TM2	IC
Site 3	TM4, TM5, TM8	IC
Site 4	TM9, EL2	EC
Site 5	TM10, TM11, TM12	IC

For each site we indicate the transmembrane helices (TM) involved in binding of CHOL and whether the site is predominantly on the intracellular (IC) or extracellular (EC) side of the lipid bilayer as revealed by the CHOL density maps (Fig 2).

**Table 3:** CHOL occupancy of site 1 and 2 as a fraction of time.

	hDAT	hSERT	hNET	dDAT
Site 1	0.50	0.89	0.58	0.55
Site 2	0.43	0.35	0.63	ND

A site was considered occupied if the center of mass (COM) of CHOL was within 8 Å of the COM of residues constituting site 1 and site 2, respectively. The residues constituting site 1 and 2 in the four systems were determined visually based on the CHOL density maps (Fig 2). ND denotes a non-detectable occupancy.

CHOL binding at site 2 was not detected in *CG-dDAT*, which seems to agree with dDAT crystal structures where this site is only occupied by CHS in 10 out of 14 structures in comparison to site 1, which is occupied by CHOL in all structures [39,40,42]. Furthermore, CHS binding at site 2 of dDAT might be more favored than CHOL due to CHS having a charged head group. However, the presence of CHOL in the crystal structures is not necessarily reflective of the CHOL interaction strength. Indeed, we observe that CHOL has the highest degree of occupancy to site 1 in hSERT (Table 3), but no CHOL molecule was observed to bind to site 1 in the hSERT crystal structures [41]. Furthermore, the CHOL site observed on the EC part of TM12 in the hSERT crystal structures is not found in the *CG-hSERT* simulation. A study performed by Ferraro et al. using a similar CG MD approach and an early homology model for hSERT (a low resolution model based on LeuT) [44], similarly identified site 1 and 2 [45].

Site 4, located on the EC part of TM9, is also of potential interest regarding CHOLs effect on the MATs function. TM9 is involved in the dimer interface of LeuT, [15] a bacterial homologue of MATs. The MATs have also been shown to form oligomers and it is therefore possible that CHOL at site 4 has a regulatory effect on oligomer formation in a manner similar to what some studies have suggested for GPCR’s [46,47]. CHOLs effect on membrane mechanical properties might also play a role in regulating oligomer formation [48,49].

Taken together, these results substantiate that CHOL at site 1 and to a lesser extent at site 2 is conserved in all human MATs. In light of experimental studies showing CHOL having a stabilizing effect on the outward-facing conformation of the transporters [34–36] and CHOL having an effect on MAT activity [29,30], we therefore hypothesize that this direct CHOL-MAT interaction at sites 1 and 2 might be functional. Note that this does not exclude a role for the other sites.

### CHOL binding sites vs. CRAC and CARC motifs

**Fig 3:**
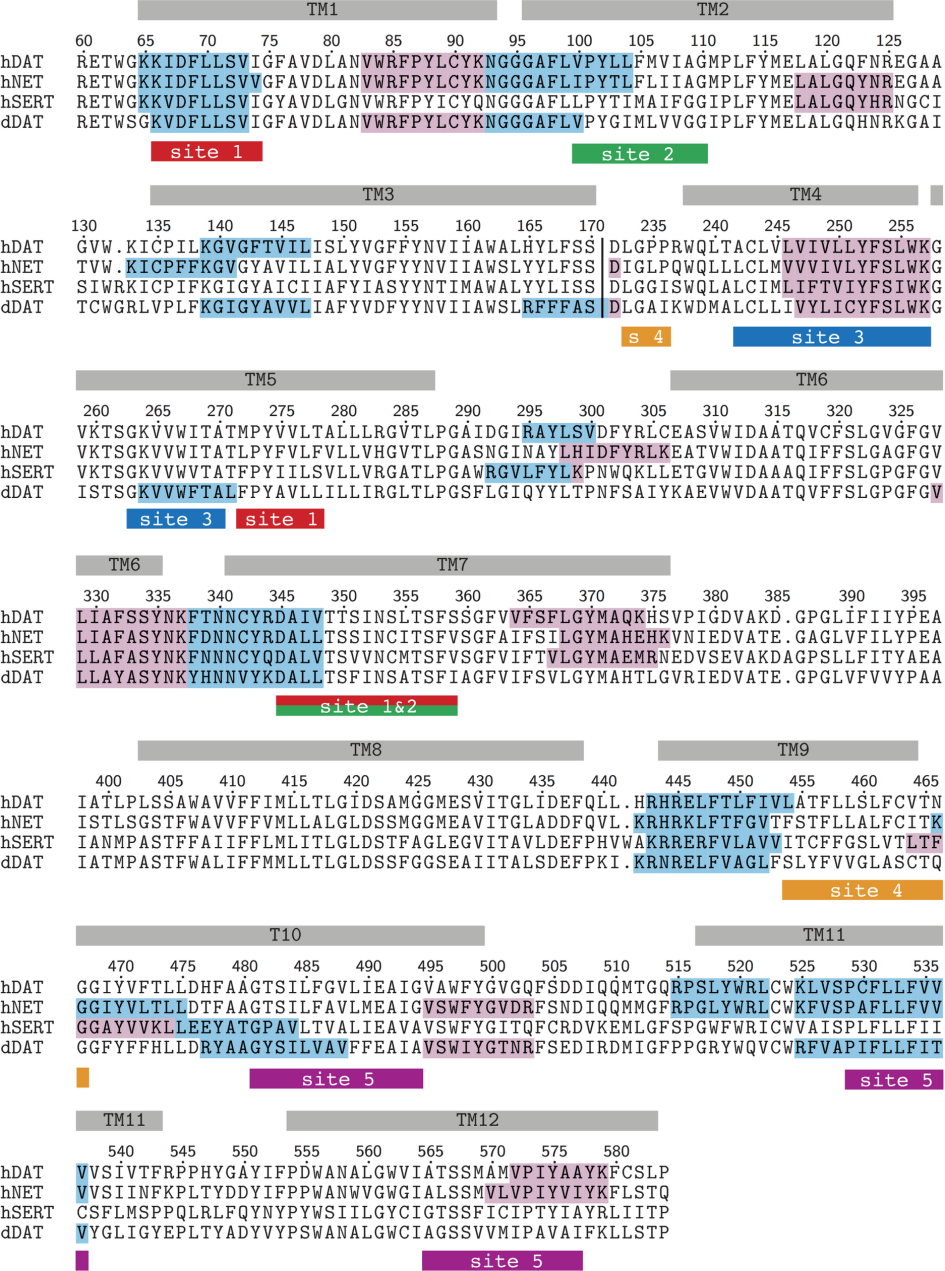
Sequence alignment of hDAT, hNET, hSERT and dDAT. hDAT numbering is indicated. CRAC (pink) and CARC (blue) CHOL binding motifs are highlighted in each transporter. The protein sections identified as CHOL binding sites (site 1–5) based on CG occupancy maps are highlighted using a different color for each site. For simplicity, the long EC loop 2 is omitted from the alignment. A vertical line between TM3 and TM4 indicates the location of the extracellular loop 2.

We evaluated whether the zones of CHOL preferential binding correlate with areas on the MATs containing conserved consensus CHOL binding motifs, CRAC (L/V-X_1−5_-Y-X_1−5_-K/R) or CARC (K/R-X_1−5_-Y/F-X_1−5_-L/V) [50–52]. We found that sites 1, 3, and 5 significantly overlap with either a CRAC or a CARC motif in most MATs (Fig 3). Site 1 contains a CARC motif in TM1a, site 3 contains a CRAC motif in TM4, and site 5 contains a CARC motif in TM11. The latter motif is not conserved for hSERT. Sites 2 and 4 do not overlap with any motif. Together, the results indicate that CARC and CRAC domains may not be a prerequisite for CHOL binding [50].

### Atomistic simulations of hDAT

AA simulations of hDAT were conducted in order to investigate if and how the co-crystallized CHOL molecules at site 1 and 2 may affect the out-to-inward conformational transition (Table 1 and Fig 1). We used a homology model of hDAT built from dDAT since no human crystal structures were available at the time the work was initiated. The very high degree of sequence identity between dDAT and hDAT (55%) assures an accurate hDAT homology model [53]. Five repeats of hDAT in an outward-facing conformation with dopamine and ions bound were each simulated for 1 μs in a POPC bilayer with and without CHOL bound at both site 1 and 2 (Table 1). Note that CHOL is only observed to interact with the protein surface within this presented study, which is in contrast to the GPCRs where CHOL has been observed to enter the substrate binding site [54].

**Fig 4:**
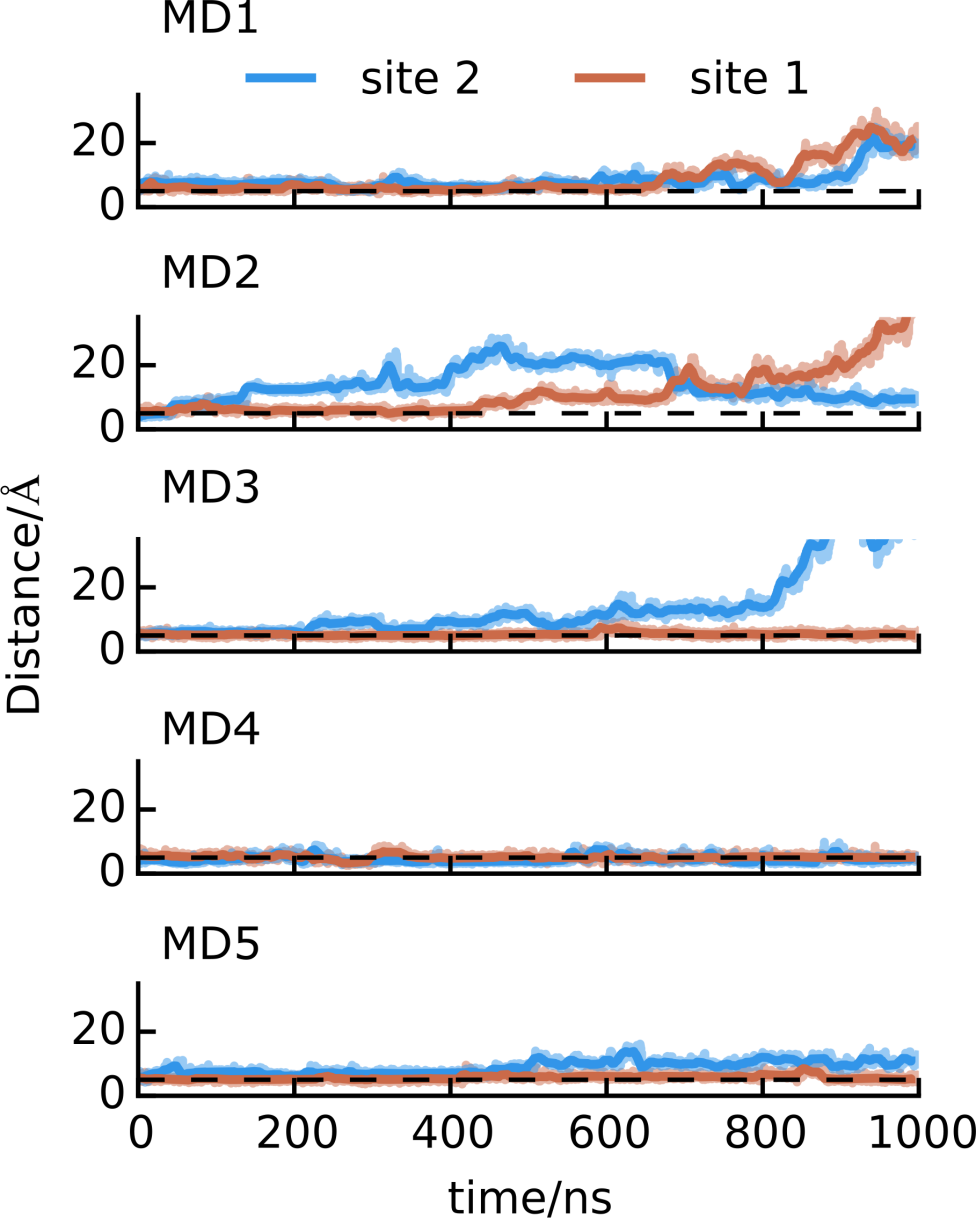
Stability of CHOL at site 1 and 2. Stability of CHOL binding at site 1 and 2 in AA MD simulations (MD1 to 5 from top to bottom). The distance monitors the separation between the COMs of a CHOL molecule and their respective binding sites. A bound CHOL molecule is defined as when the distance is shorter than 5 Å (dashed line). Note that in MD3, CHOL at site 2 diffuses further away than the graph’s cutoff (35 Å).

The CHOL molecules were relatively unstable in the *AA-hDAT-w-CHOL* simulations (Fig 4): CHOL at site 2 diffuses away in 4 out of 5 repeats (MD1-3, MD5) and twice at site 1 (MD1-2). This instability may be expected based on recent atomistic simulations, which suggests that the residence time of CHOL at protein surfaces reduces with the concentration of CHOL in the membrane bulk [55]. Nonetheless, our simulations suggest that CHOL is more stable in site 1 than in site 2.

In all the AA simulations of hDAT, the protein core of hDAT was stable within 0.25 nm root-mean-square deviation (Cα-RMSD) from the homology model (S1 Fig). Motions of the long EC loop 2 (EL2) located between TM3 and TM4 on the EC side of the transporter raises the RMSD to 0.3 nm. Per-residue root-mean-square fluctuations (RMSF) of hDAT were also very similar in all AA-hDAT simulations (S2 Fig), but in presence of CHOL, fluctuations were significantly reduced at the IC side of TM5, where CHOL at site 1 is situated. This indicates a direct stabilizing effect of CHOL on hDAT. In contrast, TM1a fluctuations are comparable and low for both system setups. This suggests that the large movement of TM1a, which is observed when comparing LeuT out and inward-facing crystal structures, is not occurring in our simulations. However, this motion of TM1a has been observed, to a lesser extent, in an earlier MD simulation of a homology model of hSERT based on the LeuT-structure [56].

### Early events of hDAT out-to-inward transition

The EC gates closed early in all AA-hDAT simulations irrespectively of whether CHOL was bound or not (S3 Fig). This occlusion of the outward-facing conformation is clearly illustrated by the formation of the R85-D476 salt bridge and the Y156-F320 aromatic lid (S3 Fig), in agreement with previous simulations of LeuT [16], hSERT [56], and hDAT [11]. In contrast, we could not identify a systematic pattern of breakage of the interaction network defining the IC gates (S3 Fig). This is in line with the recent work of Razavi et al. [10], where only the distribution of events over a very large number of hDAT simulations (> 50 x 1 μs) seemed to reflect the expected behavior (opening) rather than each simulation taken individually. Similarly, we did not observe Na^+^ release from the Na2 site, while Razavi et al. observed release in 12 out of 50 simulations. These observations also reflect apparent reduced kinetics in our simulations compared to Razavi’s, most likely resulting from the presence of a catalyzing interaction between a specialized lipid, PIP_2_, and the hDAT N-terminus [8,10,57], in the system modelled by Razavi et al. It is possible that the lack of Na^+^ release in our simulation is also due to the stochastic nature of MD simulations. An increased number of repeats could result in an increased probability for observing this event.

**Fig 5:**
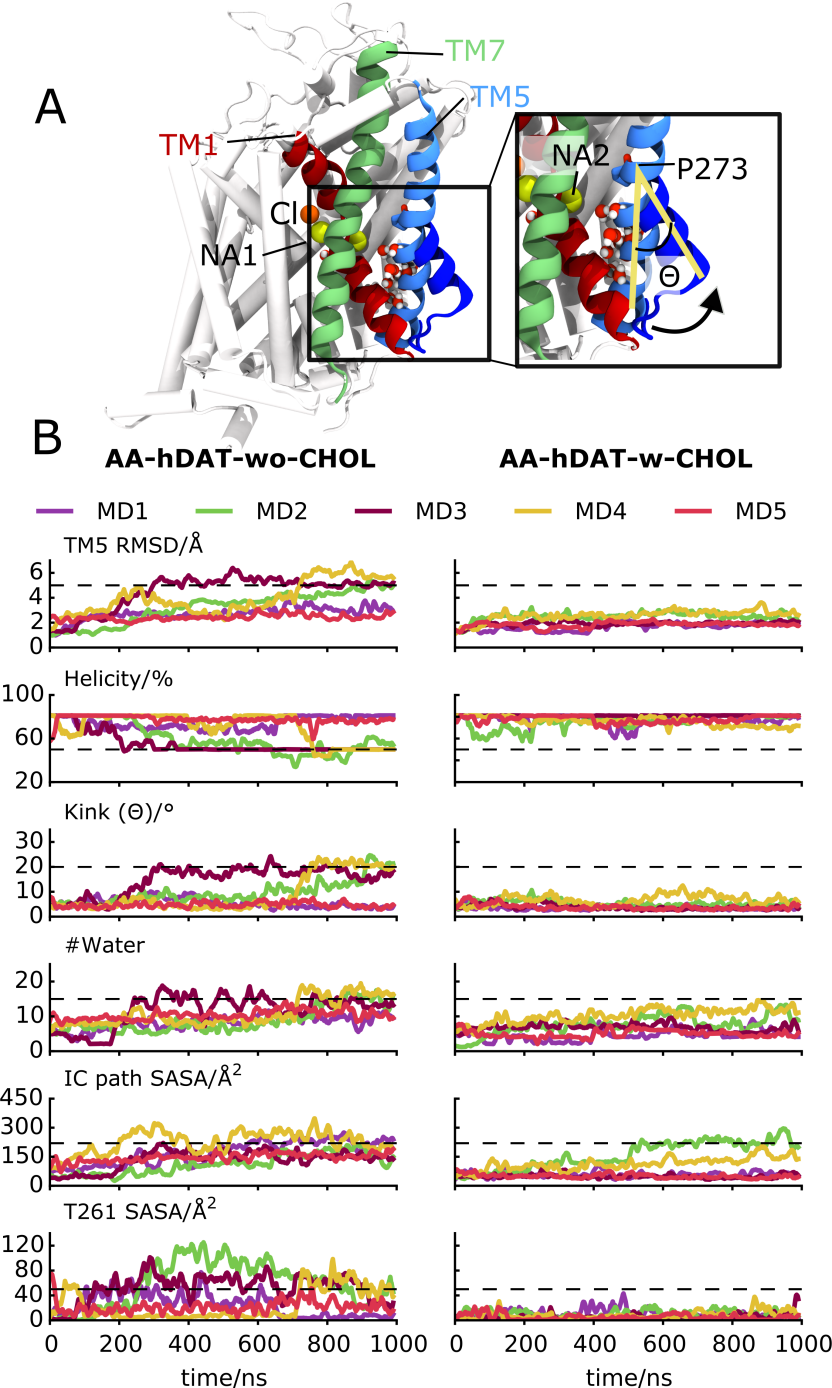
Assessment of the stabilizing effects of CHOL on the out-to-inward transition of hDAT. (A) Overlay of the last frame of two representative simulations of hDAT, one with (no transition) and one without (shows transition) CHOL. TM1, TM5, and TM7 are shown in red, green, and blue, respectively, except for the EC end of TM5 which is shown in dark blue in the hDAT structure without CHOL, thus highlighting the conformational change. The two Na^+^ ions (Na1 and Na2), Cl^-^ (Cl), and P273 are shown. (B) Systematic comparison of repeat simulations (MD1-5) with (right) and without (left) CHOL. For each simulation the time-resolved values of six parameters are monitored: RMSD of TM5, the degree of helicity of TM5 (%), the kink angle of TM5 (ϴ), the number of water molecules within 10 Å of the Na2 site, the SASA for residues proposed to line the IC exit pathway in hSERT (F69, S72, G75, G258, S262, V266, T269, F332, G425, E428, and T432), and finally the SASA for T261. The dashed lines indicate the values observed in an IN-OCC_bound_ conformation of hDAT and were visually determined from *AA-hDAT-wo-CHOL* MD2-4. The values are 5 Å for TM5 RMSD, 50% for the helicity, 20° for ϴ, 15 for #water, 220 and 50 Å^2^ for the SASA of the IC cavity and T261, respectively.

In *AA-hDAT-wo-CHOL* simulations, the events describing an early out-to-inward conformational transition (unwinding and/or outward kinking of TM1a and/or TM5 followed by increased water penetration of the IC cavity in proximity to the Na2 site) occurred in three out of five repeat simulations (MD2-4 in Fig 5). We characterized these events by monitoring the RMSD, helicity and kink of the IC end of TM5, the solvent accessible surface area (SASA) and the water count linked to the IC cavity formed between TM1a, TM5 and TM8, and finally the SASA of T261. The increase in RMSD of TM5 reflects a kink of TM5 at P273 moving its IC end away from the core of the protein and unwinding within the conserved helix-breaking motif G263(X)_9_P273 [58], similar to what has been observed for MhsT (S4 Fig) [20]. This motion of TM5 opens up a path towards the Na2 site, which quickly becomes filled with water molecules from the IC side (Fig 5A). Of potential interest, is the observation that during the preliminary opening, an interaction between S262 in TM5 and D68 is established, which we predict stabilizes the unwinding of TM5 (S3 Fig).

Interestingly, the remodeling of the IC side of TM5 led to an increase in SASA of a set of residues (F69, S72, G75, G258, S262, V266, T269, F332, G425, E428, and T432) which have previously been proposed to line the cytoplasmic pathway in hSERT (Fig 5) [59–61]. The ion in the Na2 site is expected to exit through this pore, and it is therefore predicted to become more water exposed in an inward-facing conformation. Also notable is the increased exposure of T261 at the IC end of TM5, which is a phosphorylation site dependent on the conformational state of the transporter; T261 is more phosphorylated when the transporter is in an inward-facing conformation in comparison to an outward-facing one, likely due to an increased accessibility of the residue [61–63]. Taken together, our simulations of hDAT without CHOL bound display early conformational changes that reflect the transition toward an inward-facing conformation, which is in agreement with experimental data.

### Cholesterol stabilizes hDAT in an OUT-OCC_bound_ conformation

In *AA-hDAT-w-CHOL* simulations hDAT does not display signs of the early events of the out-to-inward transition that we just described in absence of CHOL (Fig 5). In particular, TM5 neither unwinds nor kinks in any of the *AA-hDAT-w-CHOL* repeat simulations. Nevertheless, in the *AA-hDAT-w-CHOL* simulations, hDAT progresses to an occluded state (S3 Fig). We therefore propose that CHOL blocks hDAT in an OUT-OCC_bound_ conformation by preventing the key motion of TM5 in the transition to a more inward-facing conformation [22].

**Fig 6:**
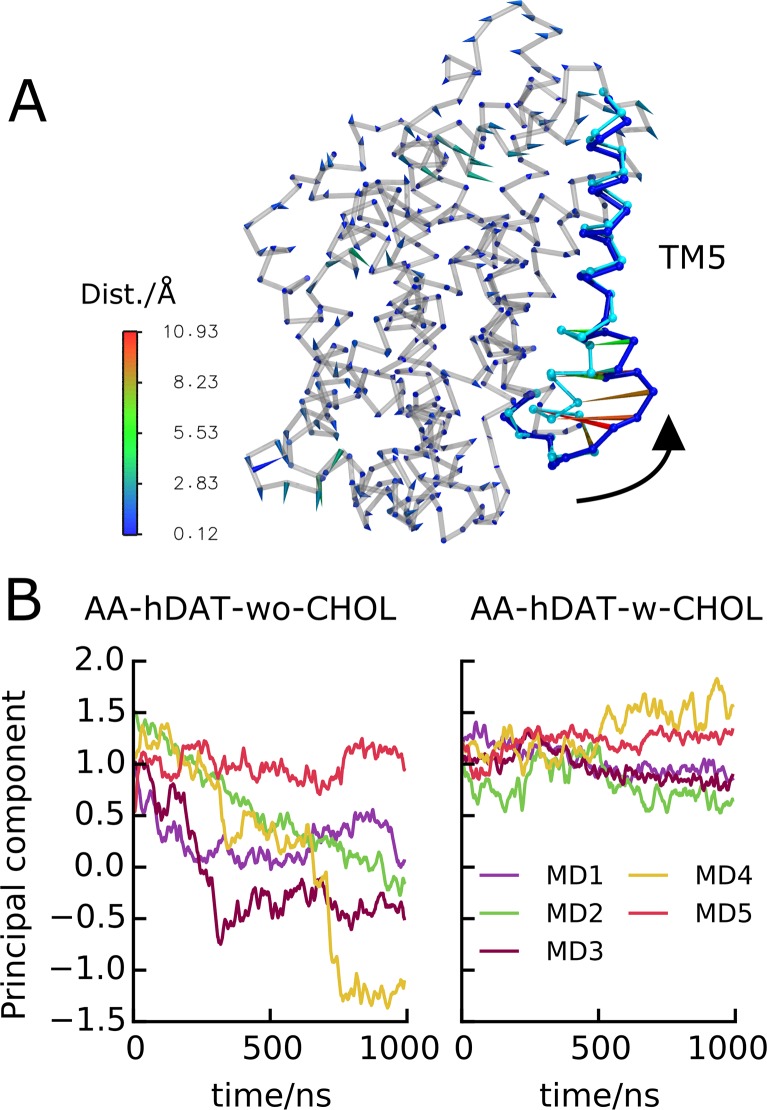
Principal component analysis of hDAT inward-opening. (A) Porcupine plot illustrating the extreme structures of the first eigenvector extracted from a PCA of *AA-hDAT-wo-CHOL* MD4. This principal component reflects the kinking and unwinding of TM5. (B) Projection of hDAT trajectories with CHOL (*AA-hDAT-w-CHOL*) and without CHOL bound (*AA-hDAT-wo-CHOL*) onto the same principal component.

The observed stability of TM5 in the *AA-hDAT-w-CHOL* systems is further underlined from the projection of all hDAT trajectories onto an eigenvector representative of TM5 motion (Fig 6). This eigenvector was extracted from a principal component analysis (PCA) of *AA-hDAT-wo-CHOL* MD4 in which the unwinding and kinking of TM5 is particularly pronounced (S5 Fig). In *AA-hDAT-w-CHOL*, TM5 does not move significantly along that eigenvector. *AA-hDAT-w-CHOL* MD2 experiences a small increase in TM5 dynamics, but the reason for this behavior was not clear from Fig 5. Visualization of the trajectory revealed that the dynamics of TM5 arises from it moving away from TM1a but without kinking or deforming, which explains the increase in SASA observed for this simulation in the absence of alteration of the TM5 conformation (Fig 5B).

In contrast, in all simulations without CHOL, hDAT explores the eigenvector widely indicating large TM5 motions, with the exception of *AA-hDAT-wo-CHOL* MD5 (Fig 6). These projections also indicate that, in the absence of CHOL, TM5 may progress differently towards a more inward-facing conformation. In MD3 and MD4, TM5 makes a rapid transition in a two-state fashion, while the transition is linear in MD2. These trends were also evident in the evolution of the RMSD, unwinding, and kinking of TM5 (Fig 5).

In most *AA-hDAT-w-CHOL* simulations we observed no water penetrating toward the Na2 site from the IC side. However, some penetration of water occurs in two simulations, MD2 and MD4 (see Fig 5), to a similar extent as observed in the *AA-hDAT-wo-CHOL* simulations. Interestingly, in the case of MD2 the water penetration nicely correlates with CHOL diffusing away from site 1, reflecting the effect of CHOL slowing down the transition when directly bound in site 1. In MD2 and MD4 the IC network encompassing E428-R445 and Y335-E428 are unstable, which would influence the degree of water penetration (S3 Fig).

Altogether these observations demonstrate that hDAT spontaneously migrates toward a more inward-facing conformation in the absence of CHOL. Furthermore, the increased stability of TM5 in the presence of CHOL, the unbinding of CHOL from site 2 and the location of site 1 at the TM1a/TM5 interface, suggests that CHOL bound to site 1 stabilizes an outward-facing conformation of hDAT and thereby prevents the transition of the transporter to an inward-facing conformation. We confirmed this hypothesis in a recent study, where we provide biochemical data to show that CHOL binding to site 1 in the serotonin transporter is key to the conformational changes necessary for transport and ligand binding [64].

### Atomistic simulations of hSERT

In light of the recent hSERT crystal structure [41], we simulated three repeats of hSERT, to confirm the sequence of events observed in the hDAT out-to-inward transition. hSERT contained serotonin and ions bound, but no CHOL (*AA-hSERT*, Table 1). In all simulations the EC gate, R104-D493, (hSERT numbering is used throughout this section) is formed and in MD1 and MD2 closure of the aromatic lid, Y178-F335, occurs (S6 Fig).

In MD1, several IC gates, Y350-E444, E444-R462, and R79-D452, break sequentially after about 0.5, 1.0, and 1.5 μs, respectively, suggesting that hSERT has initiated the out-to-inward transition (see below and S8 Fig). In MD3, R79-D452 also breaks rapidly at the beginning of the simulation to be later reformed after 0.9 μs, but no further signs of transition are observed.

**Fig 7:**
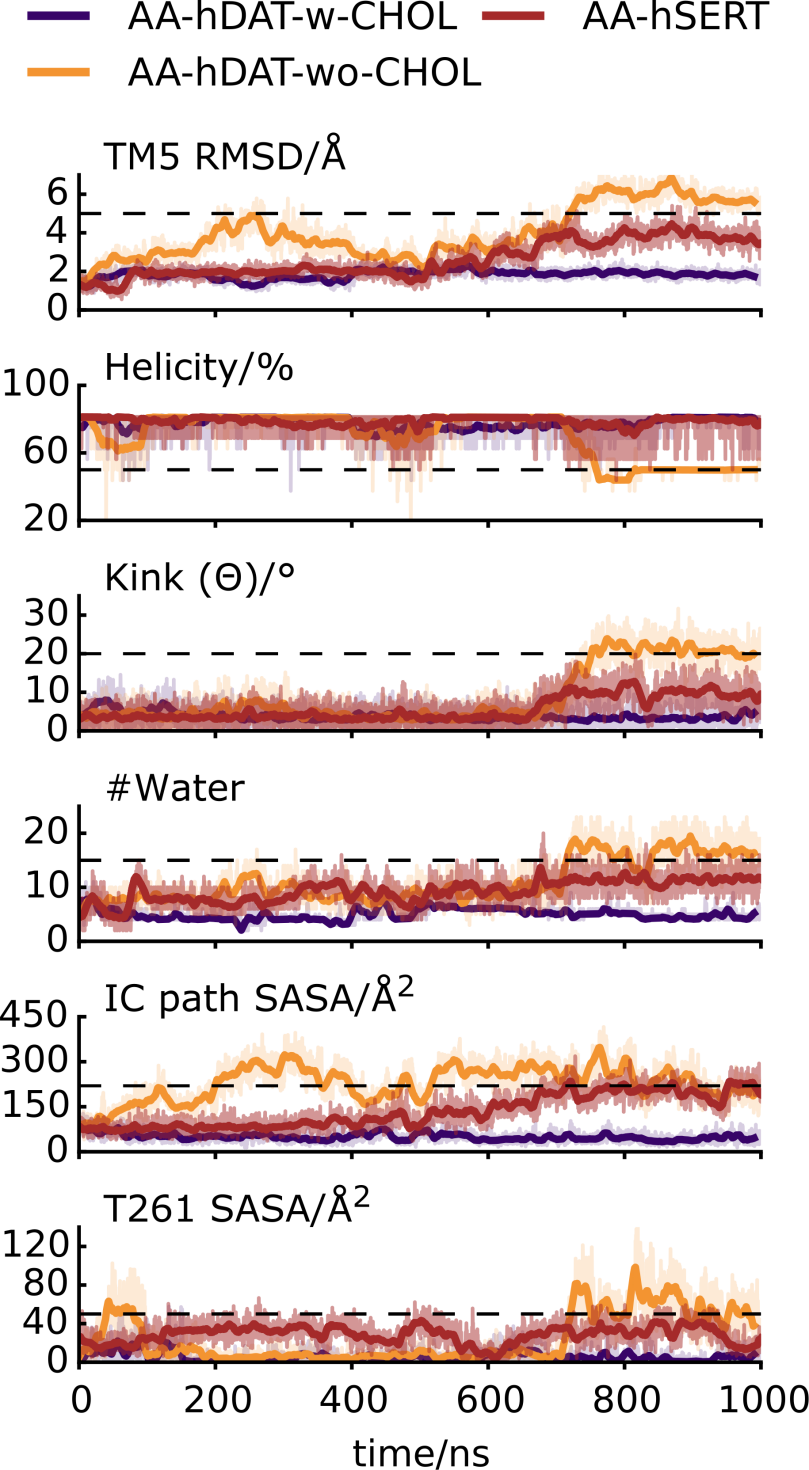
Out-to-inward conformational transition in hDAT and hSERT. Time-resolved values of six probes monitoring the early events of the out-to-inward transition for two representative hDAT simulations (*AA-hDAT-w-CHOL*: MD5, *AA-hDAT-wo-CHOL*:MD4) and a single hSERT simulation (*AA-hSERT*: MD1) is illustrated. The TM5 RMSD, degree of helicity (%), and kink angle (ϴ) were calculated for the IC half of TM5 (residues 258–273). The “#water” is the number of water molecules within 10 Å of the Na2 site. The SASA was calculated for the IC exit path (residues F69, S72, G75, G258, S262, V266, T269, F332, G425, E428, and T432), and for T261 alone. For each probe a dashed line indicates the value observed in an IN-OCC_bound_ conformation for hDAT which was visually determined. The values are 5 Å for TM5 RMSD, 50% for the helicity, 20° for ϴ, 15 for #water, 220 and 50 Å^2^ for the SASA of the IC cavity and T261, respectively. It should be noted that the hSERT simulation ran for 2 **μ**s in total, however to make comparison easier the last **μ**s of the simulation is omitted from the figure. To see the behavior of the full simulation see S7 Fig.

Sodium release and TM1a outward kinking were not observed in these hSERT simulations. However, in MD1 hSERT transitions towards a more inward-facing conformation as defined by the six parameters described above for hDAT (Fig 7 and S7 Fig). The SASA of the residues lining the IC pathway and the SASA of T276 as well as the water count, indicate a clear transition toward an inward-facing conformation of hSERT similar to what was described for hDAT (Fig 7 and Fig 5, respectively). However, in the MD1 repeat TM5 neither unwinds nor kinks to the same extent as observed for hDAT.

Preliminary work (S8 Fig) conducted on dDAT indicate that this protein engages in similar out-to-inward transitional events as reported here for hDAT and hSERT, but the simulations are much shorter (250 ns) and yet inconclusive. Collectively, these observations suggest that TM5 movement is relevant in the out-to-inward transition for DATs in multiple species.

## Discussion

In summary, we have shown that MATs share a set of CHOL binding sites among which the sites found in the crystal structures of dDAT (site 1 and 2, Fig 2) are conserved. Notable was the stable binding of CHOL at site 1 in hDAT in our AA simulations. This contact was found to immobilize the IC side of TM5, stabilizing the transporter in an outward-facing conformation thereby preventing a transition toward an inward-facing conformation. Events representative of this transition were observed in absence of CHOL bound at site 1. To our knowledge, this data represents the first evidence of an effect of CHOL on hDAT dynamics and function through a direct lipid-protein contact. Based on the high degree of conservation of site 1 and the comparable regulatory effects of CHOL on MATs [34–36], we propose that all MATs respond similarly to CHOL because of a direct lipid/protein interaction at the interface of TM1a, TM5, and TM7, which blocks the movement of TM5 and TM1a needed for the out-to-inward transition.

Notably, in all simulations of hDAT and hSERT presented here, there is no sign of an outward kinking of TM1a, as one would expect from the crystal structure of MAT’s bacterial homologue, LeuT, in an inward-facing conformation. The lack of motion of TM1a in our simulations may substantiate that TM5 unwinding, and (perhaps) kinking, is an intermediate state before TM1a outward kinking as suggested previously [20,22]. We [56] and others [8,11] have previously reported motion of TM1a in hSERT and hDAT during what was described as an out-to-inward transition, in which the movement of TM1 and the departure of Na^+^ from Na2 site were unambiguously occurring. However, a large TM1a kinking motion has mainly been reported for LeuT-based homology models [56], hDAT accelerated MD [11], and PIP_2_-enhanced inward-opening hDAT [8] simulations. The models in these approaches may be more sensitive and therefore promote TM1a outward kinking.

A comparable CHOL-protein direct interaction leading to modulation of the protein function has recently been shown for an adrenergic G-protein coupled receptor. This study was also based on computational experiments [55]. It was established that CHOL prevented the functional conformational change of a TM (TM6) using a very similar mechanism of action as reported here for hDAT. Taken together these studies demonstrate the importance of direct CHOL-protein interactions as a regulatory mechanism in biochemistry, the extent of which has yet to be fully characterized. While a considerable body of research has highlighted the importance of CHOL-rich micro domains on membrane protein function and regulation, the role of specific lipid-protein interactions may be more challenging to characterize experimentally [65–66]. In this respect computational methods show great promise as illustrated here, and we are pursuing further research along these lines.

## Methods

### Model building

As structures of dDAT and hSERT have been solved these were used after careful preparation in AA-simulations (see *protein preparation* and supporting material). hDAT and hNET models were constructed using homology modelling based on the closest evolutionary template available i.e. dDAT (PDB ID: 4XP1, sequence identities of 55% and 59%, respectively). The high degree of identity assures that the models built will be very accurate [53,67]. The template is in an outward-facing open conformation with substrate and ions bound (OUT-bound in Fig 1), and was selected based on its high resolution (2.89 Å) and because it was co-crystallized with its natural substrate, dopamine [40]. AlignMe 1.1, an online alignment tool developed specifically for membrane proteins, was used for constructing the pairwise sequence alignment of hDAT and hNET on dDAT [68].

Based on this alignment, 100 models were built for both hDAT and hNET using MODELLER v-9.15 and v-9.17, respectively [69,70]. The models were generated including loop refinement and the conserved disulfide bridge between residues 180 and 189 (residues 176 and 185 in hNET) [71,72]. The 5 models with the lowest discrete optimized protein energy (DOPE) [73] and objective function (molpdf) scores were selected for further evaluation based on ion coordination sites (the Na1, Na2, and Cl sites) [39,40,42], Ramachandran plots [74], and solvent accessibility of conserved glycosylation sites (N181, N188, and N205) [2,75].

### Protein preparation

The following section describes in detail the preparation process applied for hDAT and hSERT used in AA simulations. For the CG simulations the hNET homology model and dDAT crystal structure (PDB ID: 4XP1) were also prepared. For all crystal structures, mutations that had been introduced for crystallization reasons were corrected prior to the simulations (see S1 Table).

The hDAT model was prepared for simulations using Protein Preparation Wizard and Prime v. 3.9 in the Schrödinger Suite 2015–1 (Schrödinger, LLC). The ions situated in the Na1, Na2, and Cl sites, dopamine, and two CHOL molecules were merged from the dDAT template structure into the hDAT homology model. Hydrogens were added and the hydrogen interaction network was optimized using PROPKA [76,77] as embedded in Maestro v. 10.1.013 at pH 7.4 and from visual inspection. His225 and His373 were modelled as ε-tautomers and the rest were modelled as δ-tautomers. Glu491 was modeled protonated as it has previously been implicated in forming a Glu-Glu pair with Glu117, which may function as a conformational switch [78]. The remaining Glu and Asp residues were all modelled as charged. Finally, the structure was subjected to a restrained minimization using the OPLS2.1 force field [79] with convergence of heavy atoms set to RMSD 0.3 Å.

The hSERT crystal structure (PDB ID: 5I6X) was prepared by adding missing side chains and assigning the protonation state of titratable residues using PROPKA [76,77] as embedded in Maestro v. 10.1.013. Glu508 was protonated and all the other residues assumed a state as found at physiologic pH. The mutations I291A, T439S, and Y110A were changed back to the native residue type. The cysteine-bond between C200 and C209 was explicitly described. The position of serotonin in the transporter was taken from an earlier model [80] and the ions were located as found in the crystal structure.

### AA simulation setup

The prepared hDAT model containing dopamine (for parameters see the supporting material and S1 Appendix), ions, and CHOL was aligned to the dDAT structure (PDB ID: 4M48) from the Orientation of Protein in Membranes database [81] and used as input for the online system building tool, CHARMM-GUI [82]. ACE (acetyl) and CT2 (primary amide) caps were patched to the protein, a homogenous 1-palmitoyl-2-oleoyl-*sn*-glycero-3-phosphocholine (POPC) membrane was built and solvent was added containing 0.15 M NaCl, which also neutralizes the system. The final systems had the following dimensions; ~11 x 11 x 13 nm^3^ and contained ~ 150.000 atoms. One system contained the two co-crystallized CHOL molecules from dDAT (PDB ID: 4XP1), *AA-hDAT-w-CHOL*, and the other system contained none, *AA-hDAT-wo-CHOL*.

In the case of hSERT we used the protocol associated with the *backwards* script [83]. It consists of building a CG model, equilibrating it shortly, and transforming it back to an atomistic model system. It has the advantage of equilibrating the lipid bilayer faster. See *CG simulation setup* for the description of the building and equilibration of the CG models. After this first step the system was backmapped into an atomistic resolution using the CHARMM36 force field for both the protein and the lipids. hSERT was embedded into a POPC lipid bilayer (380 molecules) and solvated with a 0.2 M NaCl aqueous solution. The dimensions of the equilibrated box were 11.6, 11.6 and 11.3 nm in the x, y and z dimension, respectively. The simulation of this system is denoted *AA-hSERT* (Table 1).

The AA simulations were performed using Gromacs version 5.1.1 and 5.1.2 [84–86] using the CHARMM36 force field for the protein, lipids, and the charmm-modified TIP3P water parameters [87–89]. H-bonds were constrained using the LINCS algorithm [90,91] for protein and lipids and SETTLE [92] for the water, enabling the use of a 2 fs time step. The system was prepared following the protocol defined by CHARMM-GUI [82], which includes minimization using the steepest decent algorithm and six equilibration steps in which restrains on the protein and lipids are gradually decreased from 1000 kJ/mol*nm^2^[93,94]. For analysis purposes the coordinates were saved every 100 ps during the MD simulation. The temperature was kept at 310 K using a Nosé-Hoover scheme [95] and the pressure was maintained at 1 atm using a Parrinello-Rahman approach [96] with semi-isotropic scaling. A Berendsen thermo- and barostat were used for equilibration [97]. A cutoff at 12 Å was applied for the van der Waals (vdW) interactions using a switch function starting at 10 Å [98]. The cutoff for the short-range electrostatic interactions was at 12 Å and the long-range electrostatic interactions were calculated using the Particle-Mesh Ewald [99] algorithm. The protein, membrane, and aqueous phase were separated into three energy groups. The two systems, *AA-hDAT-w-chol* and *AA-hDAT-wo-chol*, were repeated 5 times and run for 1 μs each, totaling 10 μs simulation time. *AA-hSERT* was simulated 3 times for 2 μs.

### CG simulation system setup

The atomistic models for human MATs and dDAT were coarse-grained (CG) using the martinize script. The secondary structure was predicted with DSSP v. 2.0.4.[100] Each protein was placed in a POPC membrane containing 20% CHOL and solvated with 0.15 M NaCl using the INSANE tool [101]. The final systems had the box dimensions ~12x12x12 nm^3^ and contained ~15.000 CG beads. All systems had the same number of POPC and CHOL lipids; 301 and 74, respectively. In all CG systems restraints were applied on Y274 (hDAT numbering) to keep its orientation towards the core of the protein. Otherwise it was becoming exposed to the membrane, considerably altering the binding of cholesterol at site 1. This is elaborated in the supplementary material.

Each system was simulated for 50 μs with Gromacs 5.1.2 [84,85] using the Martini v.2.2 force field, [102–104] and settings recommended for simulating with the MARTINI force field [105]. The secondary structure of the protein was maintained using the ElNeDyn approach [106].The coordinates used for analysis were saved every 1 ns. The temperature was kept fixed at 310 K using the velocity rescaling method [107] with a coupling constant of 1.0 ps and the pressure was regulated at 1 bar using Parrinello-Rahman [96] semiisotropic pressure coupling and a coupling of 24 ps^-1^. The compressibility was set to 3x10^-4^ bar^-1^. For both the vdW and electrostatic interactions a 11 Å cutoff was used with the potential-shift-verlet scheme. The dielectric constant was set to 15. The electrostatics beyond the cutoff were treated by the reaction-field algorithm [108]. Before the production runs, the systems were first minimized followed by a 1μs equilibration using the Berendsen barostat [97] with a 12 ps^-1^ coupling constant and using random velocities. One repeat for each of the four proteins was carried out for 50 μs using a time step of 25 fs. Note, for the hDAT simulation, the system experienced freezing during equilibration. To avoid freezing, 10% of the water beads were replaced by anti-freeze particles during equilibration, as it has been recommended in such cases [102]. The anti-freeze particles were converted back to regular water for the production run.

### Principal component analysis

A principal component analysis (PCA) was performed on *hDAT-wo-CHOL* MD4 using GROMACS analysis tools. We restricted the analysis to the Cα atoms of residues 66–171 and 232–548. The EC loop 2 and the N and C-terminus are thereby excluded thus the focus of the analysis was on the core structure of the transporter. PCA were conducted on all simulations but we found that the first principal component of *hDAT-wo-CHOL* MD4 best represented the transition to an IN-OCC_bound_ conformation including the motion of TM5. For each simulation the protein core dynamics were projected onto the first eigenvector of this simulation. It should be noted that the cosine content for all principal components of the first eigenvectors in the systems where transition occurs is > 0.5, indicating that the system has not yet converged. However, reaching convergence is not feasible due to the large time scales of the conformational transitions of MATs.

## Supporting information

S1 Text Methods. (PDF)

S1 Appendix Dopamine parameters. (PDF)

S1 Table Overview of the proteins studied and their respective template. The residues constituting the proteins and the mutations that have been corrected are listed.
(PDF)

S1 Fig The time-resolved value of hDAT RMSD for the different repeats of the two systems without (*AA-hDAT-wo-CHOL*) and with (*AA-hDAT-w-CHOL*) CHOL. Blue represents the RMSD for the Cα-atoms within the whole protein and green represents the Cα-RMSD for hDAT excluding the N-and C terminus (residues 58–65 and 595–601), TM5 (residues 256–286), and EL2 (residues 178–236). The protein was in both cases first fitted to all hDAT Cα-atoms except the N-and C terminus, TM5 and EL2. It is clear when excluding the flexible elements in hDAT that the RMSD in all repeats for both systems quickly converges around 2–3 Å indicating an equilibrated system.
(TIF)

S2 Fig Root-mean-square fluctuation (RMSF) of hDAT Cα-atoms for the five repeats in the two systems without (*AA-hDAT-wo-CHOL*) and with (*AA-hDAT-w-CHOL*) CHOL. Highlighted in pink are all helices except TM1, TM5 and TM7, which are represented by red, blue and green bars, respectively. The numbers above the plots from 1 to 12 indicate the helix number. The largest fluctuations occur around EL2 and at the termini. However, it is also clear that TM5 fluctuates significantly more in the *AA-hDAT-wo-CHOL* system in comparison to the *AA-hDAT-w-CHOL* system. The black dotted line indicates where P276 is situated, which has a kinking and unwinding effect on the intracellular part of TM5.
(TIF)

S3 Fig hDAT extracellular and intracellular gate analysis. A) The different residue pairs are color coded to match that of B), which represent the time evolution of the distances between the pairs. Each row represents a repeat simulation and the two columns correspond to the two different systems without (*AA-hDAT-wo-CHOL*) and with (*AA-hDAT-w-CHOL*) CHOL.
(TIF)

S4 Fig hDAT and MhsT comparison. TM5 alignment of the last frame of hDAT simulated without CHOL in repeat MD4 (blue) and MhsT (magenta, PDB ID: 4US3). Both helices unwind and interact with TM1. TM5 of MhsT does not seem to kink in the middle to the same extend as observed for TM5 in hDAT. Highlighted is the conserved proline residue, P273 (hDAT numbering), for hDAT (blue) and MhsT (magenta). It is observed that the two residues are slightly displaced with respect to each other. Shown in red and black spheres are the water molecules observed in hDAT and MhsT, respectively. It is seen that the number of water molecules are approximately the same.
(TIF)

S5 Fig Principal component analysis of *AA-hDAT-wo-CHOL* MD4. A) RMSF of the first principal component shows high fluctuation around TM5. The high fluctuation corresponds to TM5 unwinding and kinking as illustrated in B), which ultimately leads to a more inward-facing conformation. The red, blue and green bars correspond to TM1, TM5 and TM7, respectively. The areas shaded light blue are the remaining transmembrane helices. The black dotted line indicates where P276 is situated, which has a kinking and unwinding effect on the intracellular part of TM5. B) The superposition of the two extremes of the first principal component. In the PCA analysis the two termini, TM12 and EL2 were omitted and only Cα atoms were considered (residues 66–170, residues 232–547).
(TIF)

S6 Fig hSERT extracellular and intracellular gate analysis. The different residue pairs measured are shown in S3A Fig, but here hSERT numbering is used. Each row represents a different simulation (MD1-3).
(TIF)

S7 Fig Three repeat simulations of hSERT without CHOL bound (*AA-hSERT*). Plotted for each repeat simulation are the time-resolved values of the six parameters; RMSD of TM5, the degree of helicity of TM5, the kink angle of TM5, the number of water molecules within 10 Å of Na2, the solvent accessible surface area (SASA) for residues proposed to make out the cytoplasmic pathway in hSERT [60] and finally SASA for T276 (hSERT numbering). T276 is proposed to be more water exposed in the inward-facing conformation. The data suggests that in MD1 hSERT is changing towards a more inward-facing conformation. However, the degree of transition is not near as high as what is observed for hDAT. In particular unwinding is barely observed.
(TIF)

S8 Fig dDAT system comparison of three repeats simulations with (*AA-hDAT-w-CHOL*) and without (*AA-hDAT-wo-CHOL*) CHOL bound to site 1. Plotted for each repeat simulation in both systems are the time-resolved values of the six parameters; RMSD of TM5, the degree of helicity of TM5, the kink angle of TM5, the number of water molecules within 10 Å of Na2, the solvent accessible surface area (SASA) for residues proposed to make out the cytoplasmic pathway in hSERT [60,109] and finally SASA for T260. T260 is proposed to be more water exposed in the inward-facing conformation of hSERT. The data suggests that the systems without CHOL experience an increased fluctuation. However, they are not as high as those observed for hDAT.
(TIF)
