# Supplementary material for: A direct interaction of cholesterol with the dopamine transporter prevents its out-to-inward transition
Source: PLoS Comput Biol. 2018 Jan 12;14(1):e1005907. doi: 10.1371/journal.pcbi.1005907 (PMC5811071; doi:10.1371/journal.pcbi.1005907)
Supplement: S1 Text — (PDF) [file pcbi.1005907.s001.pdf]

S1 Methods

Model preparation

The input primary sequence for hDAT (ID: Q01959) and hNET (ID: P23975) used were selected from Uniprot [1] and the dDAT primary sequence was extracted from the crystal structure (PDB ID: 4XP1). Therefore, the hDAT and hNET models do not contain their full N and C-termini, as these are not resolved in the dDAT template structure. In S1 Table an overview of the models and structures employed within this study is given.

Dopamine parameterization

The dopamine parameters were developed with the CHARMM general force field (CGenFF) [2-4] v. 3.0.1 using the ParamChem webserver v-1.0.0. The two aliphatic carbons and the positively charged nitrogen atom received “medium bad” penalties for their partial charges. ParamChem thus recommends conducting further validation. However, they differ only minimally when compared to previously published dopamine parameters used successfully in MD simulations [5,6] and were therefore not further optimized. The dihedral angle between the two hydroxyl groups also received a medium bad penalty and was therefore optimized with the force field toolkit (ffTK) [7] using first simulated annealing followed by a downhill simplex algorithm when fitting to the quantum mechanically calculated torsional scan. The final force constant for the dihedral was changed from 2.58 kJ/mol to 7.052 kJ/mol. The dopamine parameters are supplied in S1 Appendix.

The applied Y274 restraint in the CG systems

From CG MD simulations it was observed that Y274 (hDAT numbering) reorients to face the membrane milieu, thereby opening up a cavity between TM1, TM5, and TM7 which results in CHOL binding vertically with respect to the membrane with its hydroxyl group buried deeply within the protein cavity. This binding conformation does not reflect the binding conformation of CHOL observed in the dDAT crystal structures and in AA MD simulations. It was observed that Y274 is in close contact with T356 and that due to the coarse-graining of hDAT the distance between these two residues becomes shorter than their summed vdW radii, thereby destabilizing

Y274 which is pushed into the membrane environment. This is believed to be an artifact of the CG model. Y274 was therefore restrained to be in close proximity to T367 using bond restraints applied in all CG simulations of hNET, hDAT, hSERT, and dDAT.

AA dDAT model preparation

The dDAT structure used for the AA MD simulations presented in S9 Fig is based on the crystal structure published by Pennmatsa and co-workers in 2013 (PDB ID: 4M48) [8]. The two co-crystalized Fab domains were removed and the five point mutations present were mutated back to their respective WT amino acids. The protein was prepared using Protein Preparation Wizard (Schrödinger Suite, LLC 2012) with ions, water, nortriptyline, and CHOL at site 1 included. Missing atoms were added using Prime v. 3.1 and the protonation- and tautomeric states of relevant residues were assessed with PROPKA v. 3 [9]. The final model had E490 in its neutral form and H230 and H472 were modeled as ε-tautomers. The co-crystallized nortriptyline was then removed and dopamine was placed in the primary binding site as reported by Koldsø and co-workers [6]. The model was subjected to a minimization using the OPLS 2005 force field with a maximum heavy atom RMSD of 0.3 Å as a restraint.

AA dDAT MD simulations

Preliminary simulations conducted in our lab on a dDAT crystal structure with and without CHOL bound at site 1 also suggested a stabilizing effect of CHOL on dDAT, and stimulated the current work on the more pharmacologically relevant human homologue (hDAT). The dDAT simulations were performed on a shorter timescale and the system with bound CHOL only contained CHOL at site 1. Yet, when evaluating the same six parameters as measured for hDAT (TM5 RMSD, kink, helicity, water count, IC path solvent accessible surface area (SASA), and SASA of T261) the results show increased TM5 dynamics and water accessibility to the IC side in simulations of dDAT with CHOL in comparison to simulations of dDAT without CHOL (S9 Fig).

Two simulation setups were used: dDAT with and without CHOL bound to site 1, but both containing co-crystallized ions and dopamine. The protein was embedded in a pre-equilibrated POPC bilayer and solvated with 0.2 M NaCl. The system contained ~

85,000 atoms and had the dimensions ~ 9 x 8 x 10 nm. The system was minimized using a conjugate gradient algorithm until convergence, followed by three equilibration steps. First, the system was simulated in the NVT ensemble for 500 ps while the protein heavy atoms were restrained in all dimensions using force constants of 1000 kJ/mol/nm, allowing lipids and water molecules to adjust to the protein. Secondly, the system was simulated in the NPT ensemble for 1 ns with the same restraints, and finally, the system was simulated in the NPT ensample for 5 ns without restraints before extending the simulations to a 250 ns production run.

All simulations were performed using GROMACS 5.0.2 [10] in combination with the CHARMM36 force field. The CHOL parameters applied were from the updated C36 CHOL force field [11] and the dopamine parameters used were identical to those employed by Koldsø et al [6].

Analysis:

All analyses were performed using either in-house scripts or tools from the GROMACS analysis suite [10].

When analyzing the AA simulations, frames saved every 0.1 ns were selected. The RMSD and degree of helicity of TM5 were calculated for residues 258-273, which correspond to the cytoplasmic side of TM5. The helicity was evaluated with DSSP [12]. The TM5 kink was evaluated between the two vectors defined by the COM of each of the following three residue groups: 282-286, 273-277, and 263-267. For counting the water molecules an in-house TCL script was used, which monitors every water molecule within 10 Å of the Na^+ ion in the Na2 site. Finally, the residues within 3.5 Å of the two CHOL molecules in hDAT, were used for defining the two CHOL sites when calculating the COM distance between the sites and their respective CHOL molecules (Fig 4 in main manuscript). The SASA, was evaluated for the residues F69, S72, G75, G258, S262, V266, T269, F332, G425, E428, and T432 (Fig 5 and Fig 7 in the main manuscript) using an in-house tcl script and a probe size of 1.4 Å.

When analyzing the CG systems, frames saved every 1 ns were selected. For reporting the CHOL densities, the Volmap tool of VMD [13] was utilized by selecting the "occupancy" option and using a 0.1 nm resolution grid. Prior to the Volmap

analysis the atom sizes were changed to a radius of 2.6 Å, which corresponds to the average radius of a CG bead.

References

1. Bairoch A, Apweiler R, Wu CH, Barker WC, Boeckmann B, Ferro S, et al. The Universal Protein Resource (UniProt). Nucl Acids Res. 2005;33(suppl_1): D154-159.

2. Vanommeslaeghe K, Raman EP, MacKerell AD. Automation of the CHARMM General Force Field (CGenFF) II: Assignment of Bonded Parameters and Partial Atomic Charges. J Chem Inf Model. 2012;52(12): 3155-3168.

3. Vanommeslaeghe K, Hatcher E, Acharya C, Kundu S, Zhong S, Shim J, et al. CHARMM general force field: A force field for drug-like molecules compatible with the CHARMM all-atom additive biological force fields. J Comput Chem. 2010;31(4): 671-690.

4. Vanommeslaeghe K, MacKerell AD. Automation of the CHARMM General Force Field (CGenFF) I: Bond Perception and Atom Typing. J Chem Inf Model. 2012;52(12): 3144-3154.

5. Cheng MH, Bahar I. Molecular mechanism of dopamine transport by human dopamine transporter. Structure. 2015;23(11): 2171-2181.

6. Koldsø H, Christiansen AB, Sinning S, Schiøtt B. Comparative Modeling of the Human Monoamine Transporters: Similarities in Substrate Binding. ACS Chem Neurosci. 2013;4(2): 295-309.

7. Mayne CG, Saam J, Schulten K, Tajkhorshid E, Gumbart JC. Rapid parameterization of small molecules using the force field toolkit. J Comput Chem. 2013;34(32): 2757-2770.

8. Penmatsa A, Wang KH, Gouaux E. X-ray structure of dopamine transporter elucidates antidepressant mechanism. Nature. 2013;503(7474): 85-90.

9. Søndergaard CR, Olsson MH, Rostkowski M, Jensen JH. Improved treatment of ligands and coupling effects in empirical calculation and rationalization of p K a values. Journal of Chemical Theory and Computation. 2011;7(7): 2284-2295.

10. Lindahl E, Hess B, van der Spoel D. GROMACS 3.0: a package for molecular simulation and trajectory analysis. J Mol Model. 2001;7: 306-317.

11. Klauda JB, Venable RM, Freites JA, O'Connor JW, Tobias DJ, Mondragon-Ramirez C, et al. Update of the CHARMM All-Atom Additive Force Field for Lipids: Validation on Six Lipid Types. J Phys Chem B. 2010;114(23): 7830-7843.

12. Kabsch W, Sander C. Dictionary of protein secondary structure: pattern recognition of hydrogen-bonded and geometrical features. Biopolymers. 1983;22(12): 2577-2637.

13. Humphrey W, Dalke A, Schulten K. VMD: Visual Molecular Dynamics. J Mol Graphics. 1996;14(1): 33-38.
