# Supplementary material for: A direct interaction of cholesterol with the dopamine transporter prevents its out-to-inward transition
Source: PLoS Comput Biol. 2018 Jan 12;14(1):e1005907. doi: 10.1371/journal.pcbi.1005907 (PMC5811071; doi:10.1371/journal.pcbi.1005907)
Supplement: S1 Appendix — (PDF) [file pcbi.1005907.s002.pdf]

```
* Dopamine parameters used in:

* A Direct Interaction of Cholesterol with the Dopamine Transporter Prevents its Out-
* to-Inward Transition

*Talia Zeppelin, Lucy Kate Ladefoged, Steffen Sinning, Xavier Periole, Birgit Schiøtt

*
* Toppar  stream file generated by
* CHARMM General Force Field (CGenFF) program version 1.0.0
* For use with CGenFF version 3.0.1
*

read rtf card append
* Topologies generated by
* CHARMM General Force Field (CGenFF) program version 1.0.0
*
36 1

! "penalty" is the highest penalty score of the associated parameters.
! Penalties lower than 10 indicate the analogy is fair; penalties between 10
! and 50 mean some basic validation is recommended; penalties higher than
! 50 indicate poor analogy and mandate extensive validation/optimization.

RESI DOPA    1.000 ! param penalty=  41.200 ; charge penalty=  43.357
GROUP            ! CHARGE   CH_PENALTY
ATOM C1     CG2R61 -0.115 !    5.376
ATOM C2     CG2R61 -0.003 !    6.973
ATOM C3     CG2R61 -0.117 !    5.376
ATOM C4     CG2R61  0.113 !    0.000
ATOM C5     CG2R61  0.106 !    0.000
ATOM C6     CG2R61 -0.107 !    0.000
ATOM O1     OG311  -0.530 !    0.000
ATOM O2     OG311  -0.530 !    0.000
ATOM C7     CG321  -0.131 !   43.357
ATOM C8     CG324   0.080 !   42.317
ATOM N1     NG3P3  -0.301 !   17.712
ATOM H1     HGR61   0.115 !    0.000
ATOM H2     HGR61   0.115 !    0.000
ATOM H3     HGR61   0.115 !    0.000
ATOM H4     HGP2    0.330 !    0.000
ATOM H5     HGP2    0.330 !    0.000
ATOM H6     HGP2    0.330 !    0.000
ATOM H7     HGP1    0.420 !    0.000
ATOM H8     HGP1    0.420 !    0.000
ATOM H9     HGA2    0.090 !    0.000
ATOM H10    HGA2    0.090 !    0.000
ATOM H11    HGA2    0.090 !    2.455
ATOM H12    HGA2    0.090 !    2.455
```

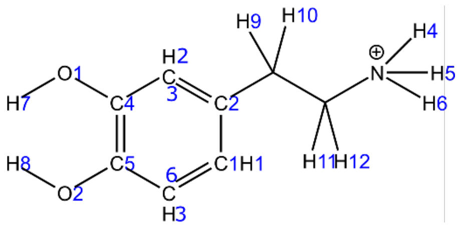


```
BOND C1   C2
BOND C1   C6
BOND C1   H1
BOND C2   C3
BOND C2   C7
BOND C3   C4
BOND C3   H2
BOND C4   C5
BOND C4   O1
BOND C5   C6
BOND C5   O2
BOND C6   H3
BOND O1   H7
```

```
BOND O2   H8
BOND C7   C8
BOND C7   H9
BOND C7   H10
BOND C8   N1
BOND C8   H11
BOND C8   H12
BOND N1   H4
BOND N1   H5
BOND N1   H6

END

read param card flex append
* Parameters generated by analogy by
* CHARMM General Force Field (CGenFF) program version 1.0.0
*

! Penalties lower than 10 indicate the analogy is fair; penalties between 10
! and 50 mean some basic validation is recommended; penalties higher than
! 50 indicate poor analogy and mandate extensive validation/optimization.

BONDS

ANGLES
CG2R61 CG321  CG324    51.80    107.50 ! Struct , from CG2R61 CG321 CG314, penalty=
0.6

DIHEDRALS
OG311  CG2R61 CG2R61 OG311      7.0520  2   180.00 ! Struct , from NG311 CG2R61
CG2R61 OG3R60, penalty= 41.2 EDITED! prevously K=2.58 according to paramchem
CG2R61 CG2R61 CG321  CG324      0.2300  2   180.00 ! Struct , from CG2R61 CG2R61
CG321 CG314, penalty= 0.6
CG2R61 CG321  CG324  NG3P3      0.2000  3     0.00 ! Struct , from NG3P3 CG314 CG321
CG2R61, penalty= 4
CG2R61 CG321  CG324  HGA2       0.0400  3     0.00 ! Struct , from CG2R61 CG321 CG321
HGA2, penalty= 1

IMPROPERS

END
RETURN
```
