# Supplementary material for: A direct interaction of cholesterol with the dopamine transporter prevents its out-to-inward transition
Source: PLoS Comput Biol. 2018 Jan 12;14(1):e1005907. doi: 10.1371/journal.pcbi.1005907 (PMC5811071; doi:10.1371/journal.pcbi.1005907)
Supplement: S1 Table — The residues constituting the proteins and the mutations that have been corrected are listed. (PDF) [file pcbi.1005907.s003.pdf]

S1 Table : Overview of the proteins studied and their respective template.

Protein	hDAT	hNET	hSERT	dDAT
Residues	58-601	54-598	74-617	25-161,203-600
Mutations corrected			I291A, T439S, A554C, A580C, Y110A	V74A, L415A
PDB ID	4XP1 (template)	4XP1 (template)	5I6X	4XP1

The residues constituting the proteins and the mutations that have been corrected are listed.
