# Supplementary figures and images for: A direct interaction of cholesterol with the dopamine transporter prevents its out-to-inward transition
Source: PLoS Comput Biol. 2018 Jan 12;14(1):e1005907. doi: 10.1371/journal.pcbi.1005907 (PMC5811071; doi:10.1371/journal.pcbi.1005907)

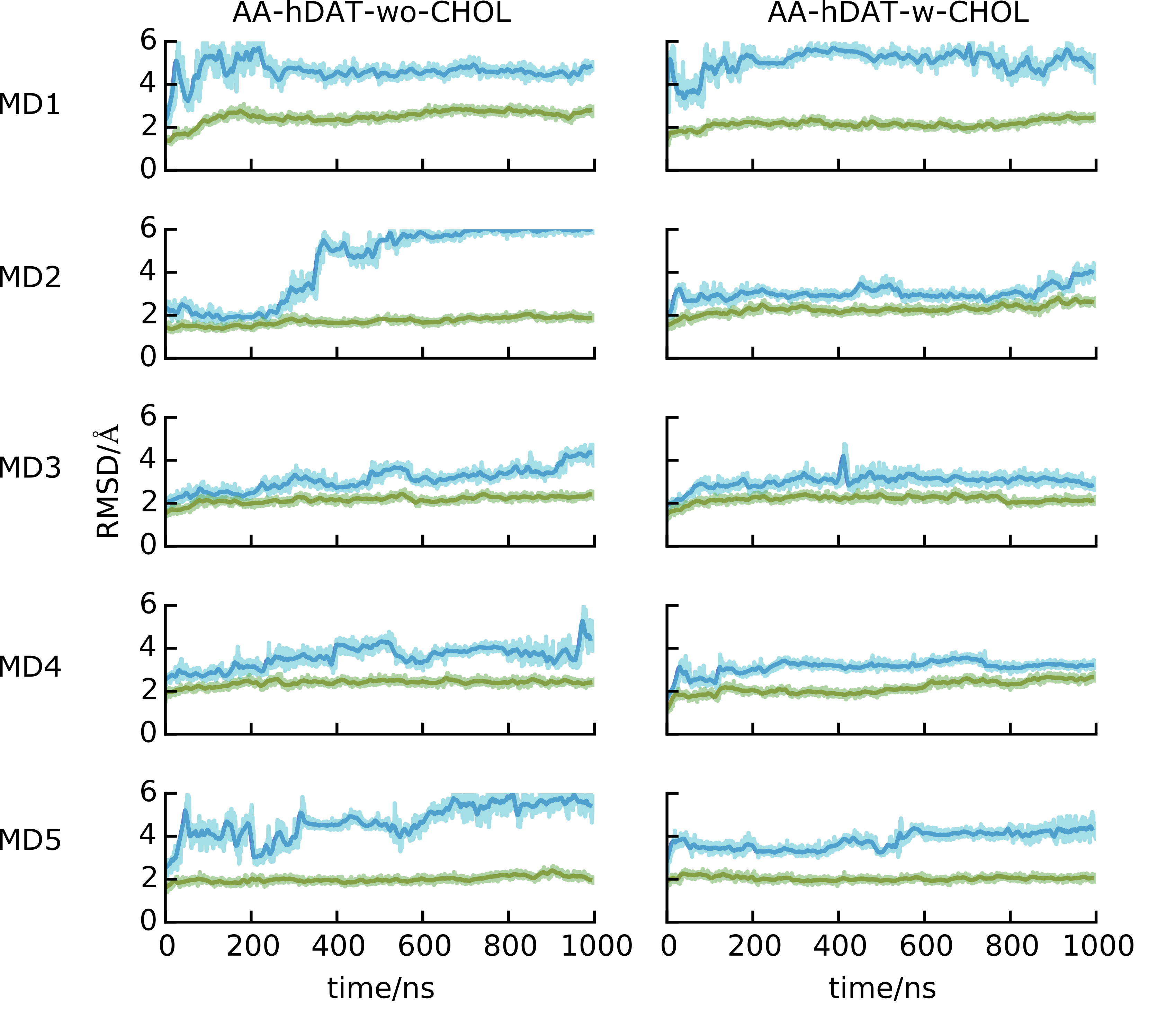

Supplement: S1 Fig — Blue represents the RMSD for the Cα-atoms within the whole protein and green represents the Cα-RMSD for hDAT excluding the N-and C terminus (residues 58–65 and 595–601), TM5 (residues 256–286), and EL2 (residues 178–236). The protein was in both cases first fitted to all hDAT Cα-atoms except the N-and C terminus, TM5 and EL2. It is clear when excluding the flexible elements in hDAT that the RMSD in all repeats for both systems quickly converges around 2–3 Å indicating an equilibrated system. (TIF) [file pcbi.1005907.s004.tif]

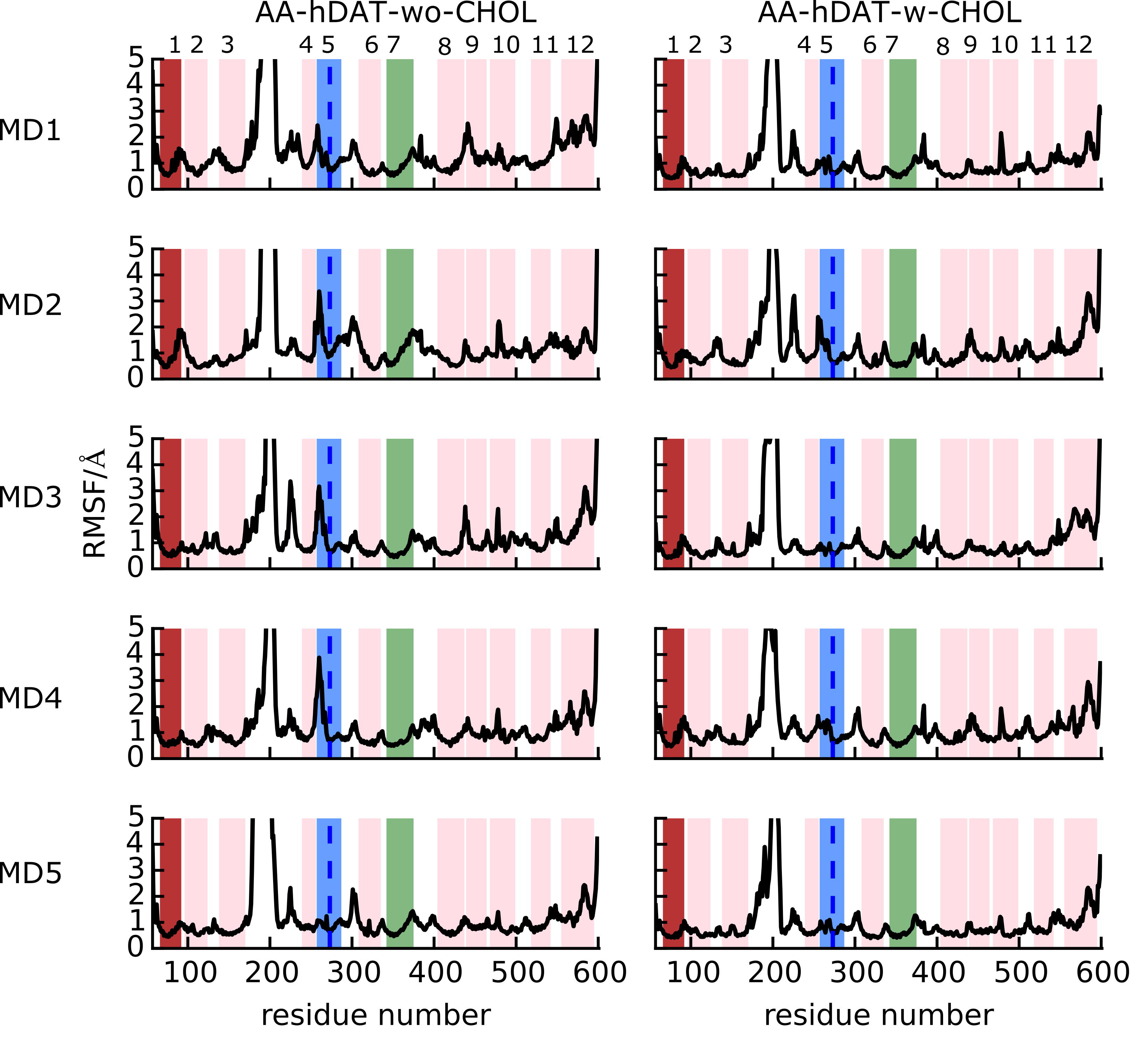

Supplement: S2 Fig — Highlighted in pink are all helices except TM1, TM5 and TM7, which are represented by red, blue and green bars, respectively. The numbers above the plots from 1 to 12 indicate the helix number. The largest fluctuations occur around EL2 and at the termini. However, it is also clear that TM5 fluctuates significantly more in the AA-hDAT-wo-CHOL system in comparison to the AA-hDAT-w-CHOL system. The black dotted line indicates where P276 is situated, which has a kinking and unwinding effect on the intracellular part of TM5. (TIF) [file pcbi.1005907.s005.tif]

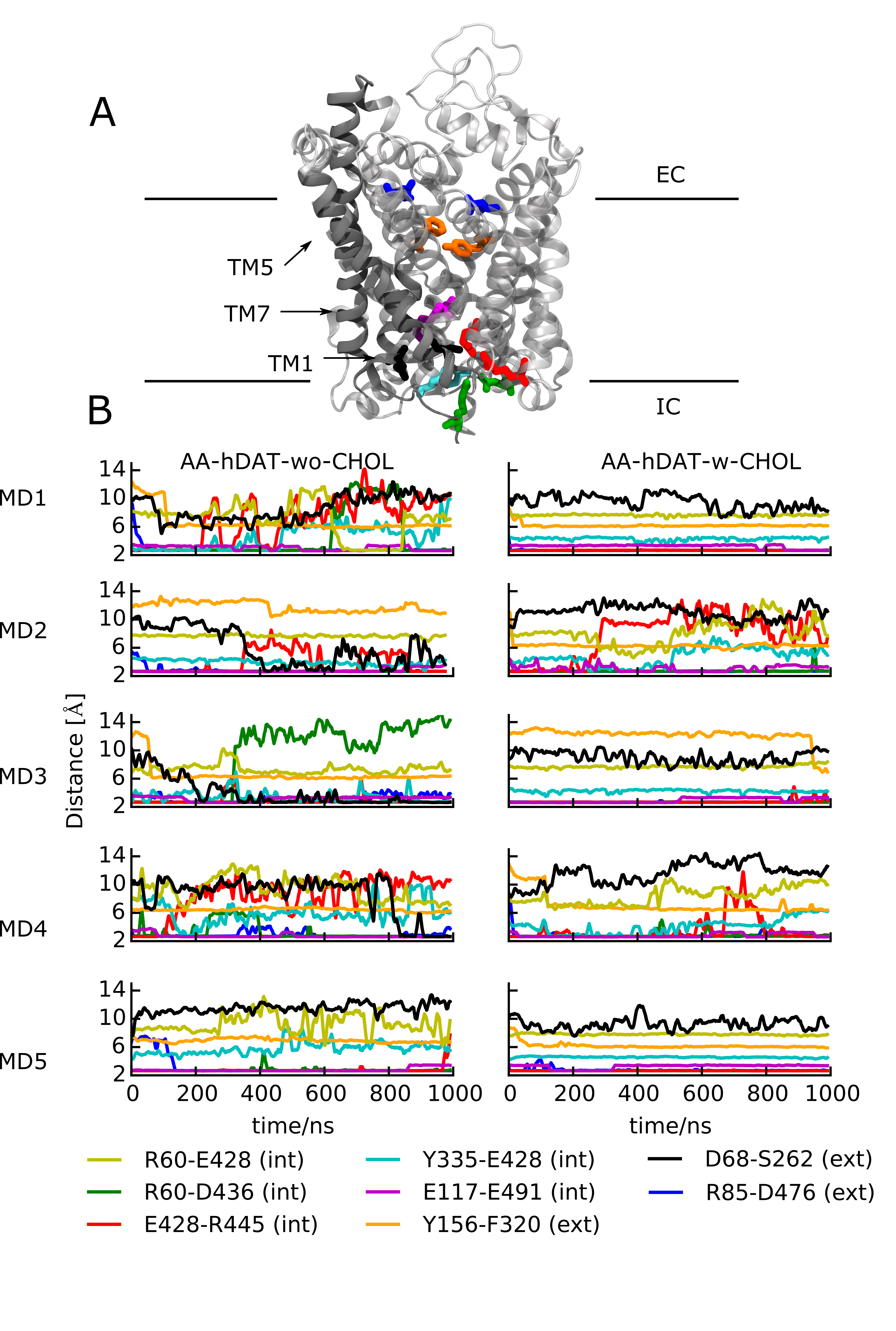

Supplement: S3 Fig — A) The different residue pairs are color coded to match that of B), which represent the time evolution of the distances between the pairs. Each row represents a repeat simulation and the two columns correspond to the two different systems without (AA-hDAT-wo-CHOL) and with (AA-hDAT-w-CHOL) CHOL. (TIF) [file pcbi.1005907.s006.tif]

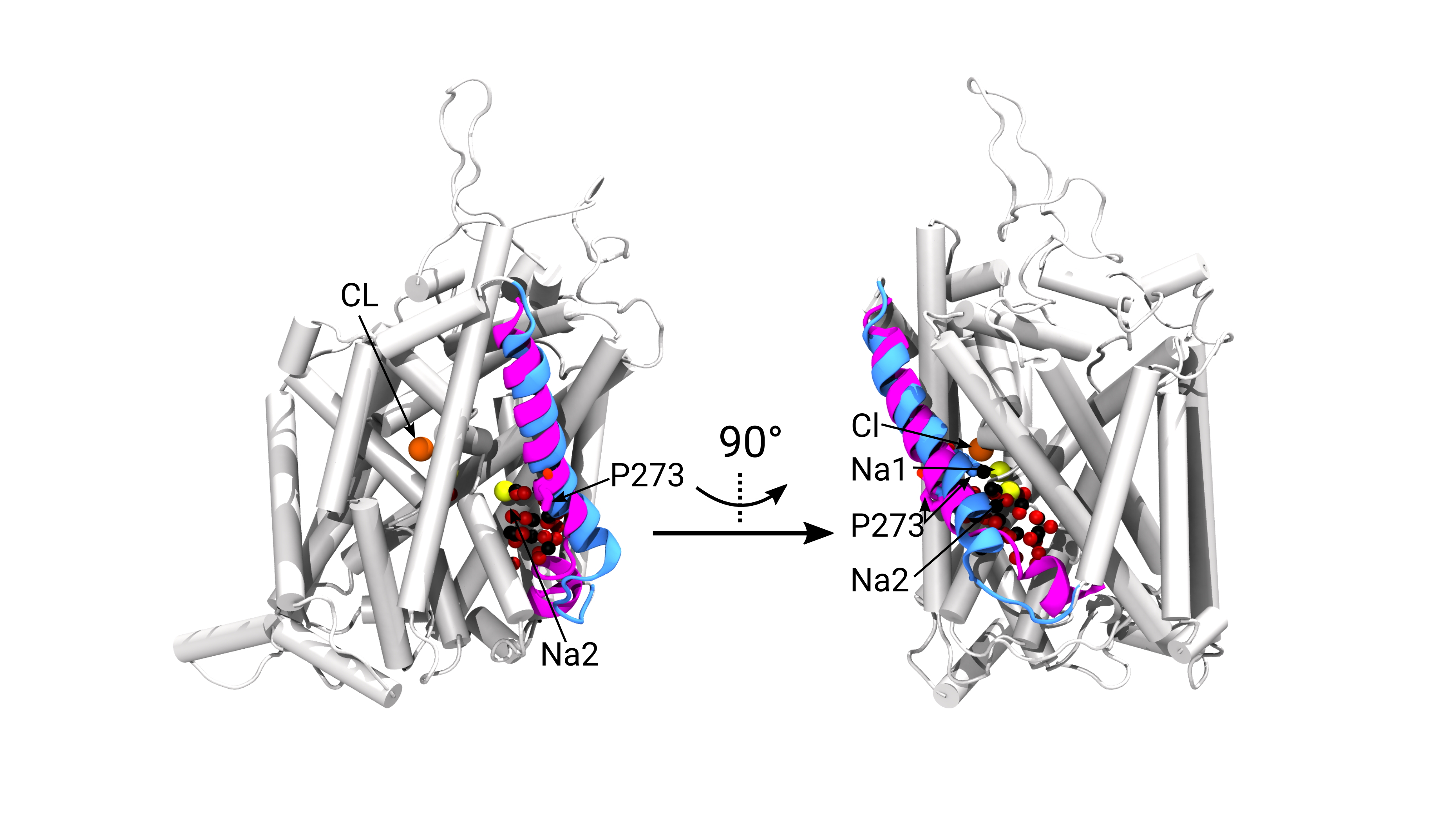

Supplement: S4 Fig — TM5 alignment of the last frame of hDAT simulated without CHOL in repeat MD4 (blue) and MhsT (magenta, PDB ID: 4US3). Both helices unwind and interact with TM1. TM5 of MhsT does not seem to kink in the middle to the same extend as observed for TM5 in hDAT. Highlighted is the conserved proline residue, P273 (hDAT numbering), for hDAT (blue) and MhsT (magenta). It is observed that the two residues are slightly displaced with respect to each other. Shown in red and black spheres are the water molecules observed in hDAT and MhsT, respectively. It is seen that the number of water molecules are approximately the same. (TIF) [file pcbi.1005907.s007.tif]

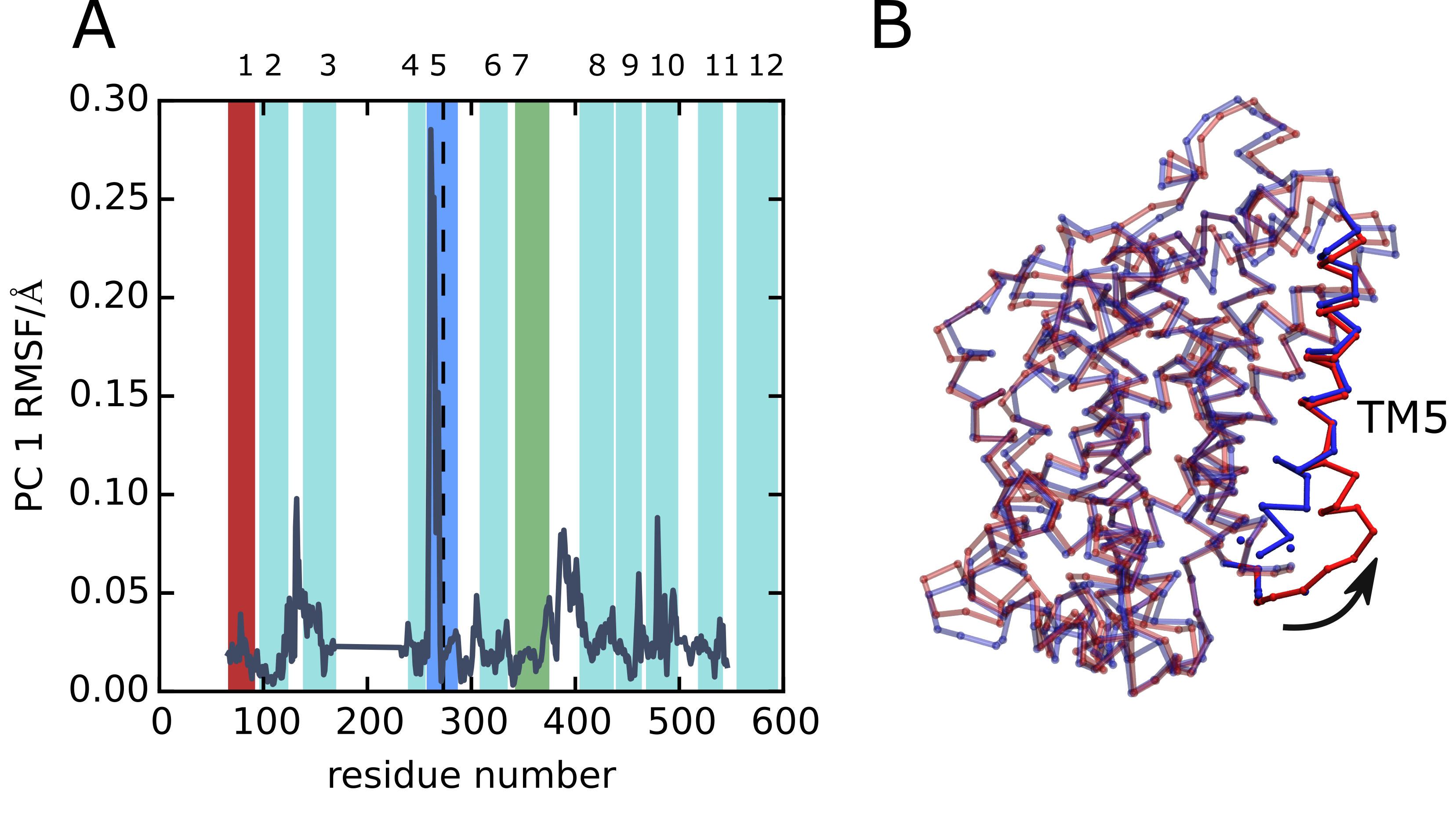

Supplement: S5 Fig — A) RMSF of the first principal component shows high fluctuation around TM5. The high fluctuation corresponds to TM5 unwinding and kinking as illustrated in B), which ultimately leads to a more inward-facing conformation. The red, blue and green bars correspond to TM1, TM5 and TM7, respectively. The areas shaded light blue are the remaining transmembrane helices. The black dotted line indicates where P276 is situated, which has a kinking and unwinding effect on the intracellular part of TM5. B) The superposition of the two extremes of the first principal component. In the PCA analysis the two termini, TM12 and EL2 were omitted and only Cα atoms were considered (residues 66–170, residues 232–547). (TIF) [file pcbi.1005907.s008.tif]

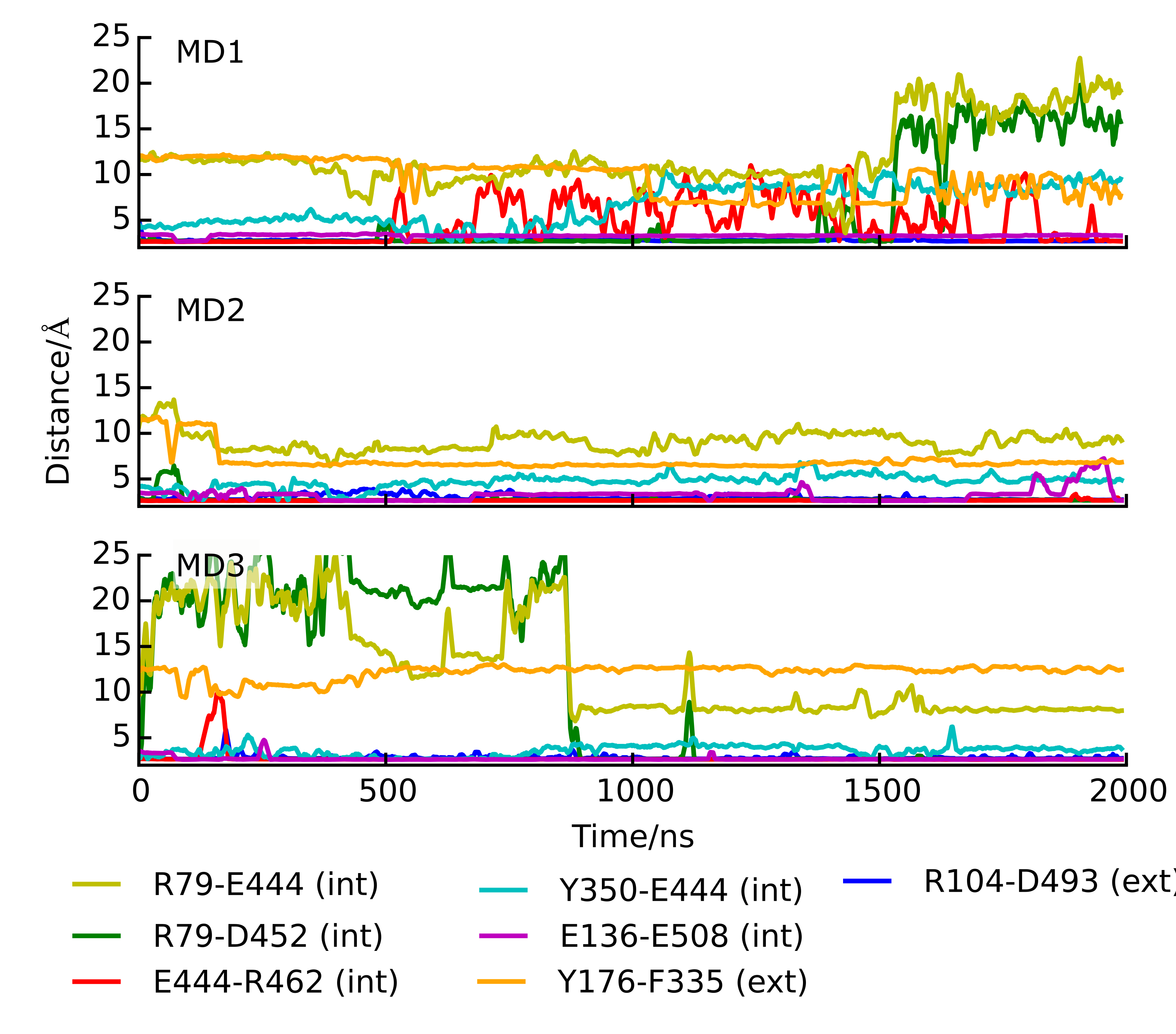

Supplement: S6 Fig — The different residue pairs measured are shown in S3A Fig, but here hSERT numbering is used. Each row represents a different simulation (MD1-3). (TIF) [file pcbi.1005907.s009.tif]

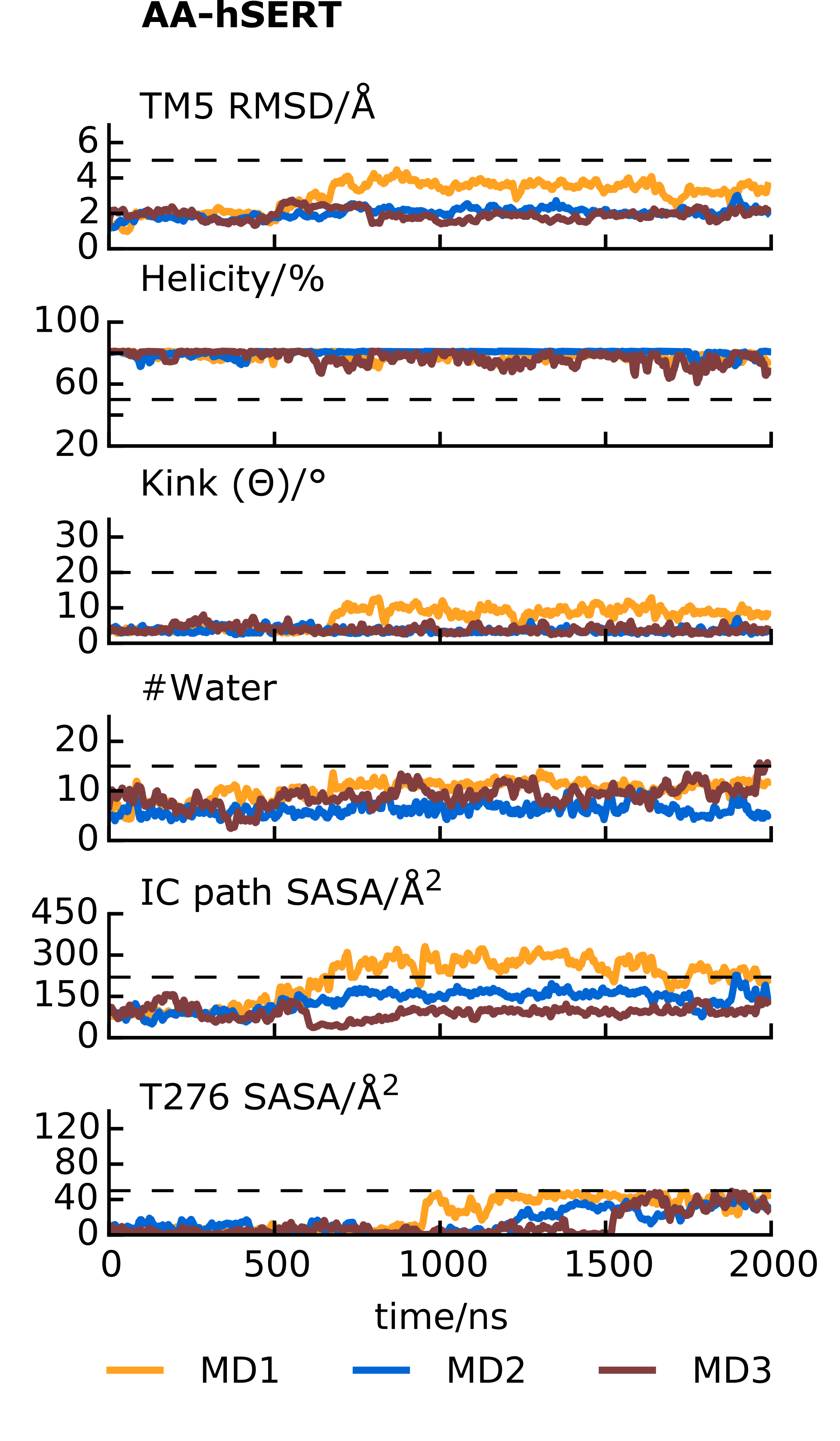

Supplement: S7 Fig — Plotted for each repeat simulation are the time-resolved values of the six parameters; RMSD of TM5, the degree of helicity of TM5, the kink angle of TM5, the number of water molecules within 10 Å of Na2, the solvent accessible surface area (SASA) for residues proposed to make out the cytoplasmic pathway in hSERT [60] and finally SASA for T276 (hSERT numbering). T276 is proposed to be more water exposed in the inward-facing conformation. The data suggests that in MD1 hSERT is changing towards a more inward-facing conformation. However, the degree of transition is not near as high as what is observed for hDAT. In particular unwinding is barely observed. (TIF) [file pcbi.1005907.s010.tif]

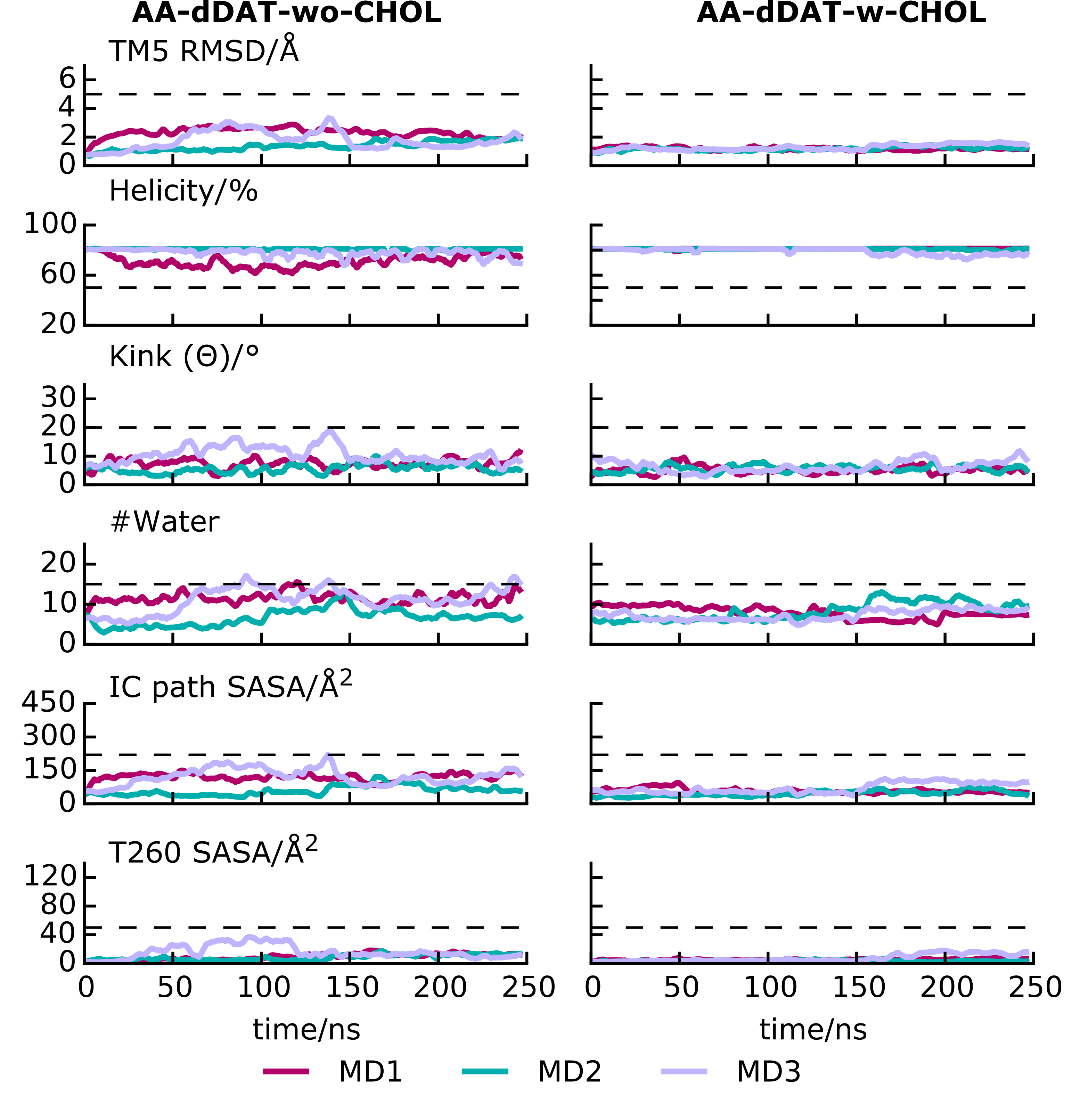

Supplement: S8 Fig — Plotted for each repeat simulation in both systems are the time-resolved values of the six parameters; RMSD of TM5, the degree of helicity of TM5, the kink angle of TM5, the number of water molecules within 10 Å of Na2, the solvent accessible surface area (SASA) for residues proposed to make out the cytoplasmic pathway in hSERT [60,109] and finally SASA for T260. T260 is proposed to be more water exposed in the inward-facing conformation of hSERT. The data suggests that the systems without CHOL experience an increased fluctuation. However, they are not as high as those observed for hDAT. (TIF) [file pcbi.1005907.s011.tif]
